# Anthelmintic drug actions in resistant and susceptible *C. elegans* revealed by electrophysiological recordings in a multichannel microfluidic device

**DOI:** 10.1016/j.ijpddr.2018.10.003

**Published:** 2018-10-30

**Authors:** Janis C. Weeks, Kristin J. Robinson, Shawn R. Lockery, William M. Roberts

**Affiliations:** Institute of Neuroscience, University of Oregon, 1254 University of Oregon, Eugene, OR, 97403-1254, USA

**Keywords:** Anthelmintic drug, *C. elegans*, Drug screening, Electrophysiology, Microfluidics, Pharyngeal pumping, 5HT, 5-hydroxytryptamine (serotonin), AChR, acetylcholine receptor, C_1/2_, concentration that causes half-maximal activation, CF, cumulative fraction of pumps, *CF*_*50*_, time at which 50% of the pumps have occurred after switching perfusate, CGC, *Caenorhabditis* Genetics Center, E spike (in EPG recording), onset of muscle contraction during a pump, EC_50_, drug concentration at half-maximal effect, EPG, electropharyngeogram, GABA-R, γ-aminobutyric acid receptor, GluCl, glutamate-gated chloride channel, IC, ion channel, IPI, inter-pump interval, IVM, ivermectin, LB, Luria-Bertani medium, L-AChR, levamisole-sensitive acetylcholine receptor, LEV, levamisole, M9, M9 buffer, M9-5HT, M9 buffer containing 10 mM 5HT, NGM, nematode growth medium, NTR, neurotransmitter receptor, O.D._600_, optical density at 600 nm, OP50, strain of *E. coli* used to feed *C. elegans* in the laboratory, PDMS, polydimethylsiloxane, PPZ, piperazine, R spike (in EPG recording), muscle relaxation at the end of a pump, RMS, root mean square, *R*_*seal*_, seal resistance between the worm's body and the microfluidic channel walls, SAR, structure–activity relationship, *t*_1/2_, time until half-maximal value is reached

## Abstract

Many anthelmintic drugs used to treat parasitic nematode infections target proteins that regulate electrical activity of neurons and muscles: ion channels (ICs) and neurotransmitter receptors (NTRs). Perturbation of IC/NTR function disrupts worm behavior and can lead to paralysis, starvation, immune attack and expulsion. Limitations of current anthelmintics include a limited spectrum of activity across species and the threat of drug resistance, highlighting the need for new drugs for human and veterinary medicine. Although ICs/NTRs are valuable anthelmintic targets, electrophysiological recordings are not commonly included in drug development pipelines. We designed a medium-throughput platform for recording electropharyngeograms (EPGs)—the electrical signals emitted by muscles and neurons of the pharynx during pharyngeal pumping (feeding)—in *Caenorhabditis elegans* and parasitic nematodes. The current study in *C. elegans* expands previous work in several ways. Detecting anthelmintic bioactivity in drugs, compounds or natural products requires robust, sustained pharyngeal pumping under baseline conditions. We generated concentration-response curves for stimulating pumping by perfusing 8-channel microfluidic devices (chips) with the neuromodulator serotonin, or with *E. coli* bacteria (*C. elegans*’ food in the laboratory). Worm orientation in the chip (head-first vs. tail-first) affected the response to *E. coli* but not to serotonin. Using a panel of anthelmintics—ivermectin, levamisole and piperazine—targeting different ICs/NTRs, we determined the effects of concentration and treatment duration on EPG activity, and successfully distinguished control (N2) and drug-resistant worms (*avr-14; avr-15; glc-1, unc-38* and *unc-49*). EPG recordings detected anthelmintic activity of drugs that target ICs/NTRs located in the pharynx as well as at extra-pharyngeal sites. A *bus-8* mutant with enhanced permeability was more sensitive than controls to drug treatment. These results provide a useful framework for investigators who would like to more easily incorporate electrophysiology as a routine component of their anthelmintic research workflow.

## Highlights

• Microfluidic electropharyngeogram (EPG) recordings reveal pharyngeal pumping.
• *E. coli* or serotonin evoke sustained pumping in *C. elegans* in microfluidic chips.
• Anthelmintic drugs inhibit pumping in a concentration- and time-dependent manner.
• EPG recordings distinguish susceptible from drug-resistant worms.
• The 8-channel EPG platform is a useful tool for anthelmintic research.

## Graphical abstract

**Figure:**
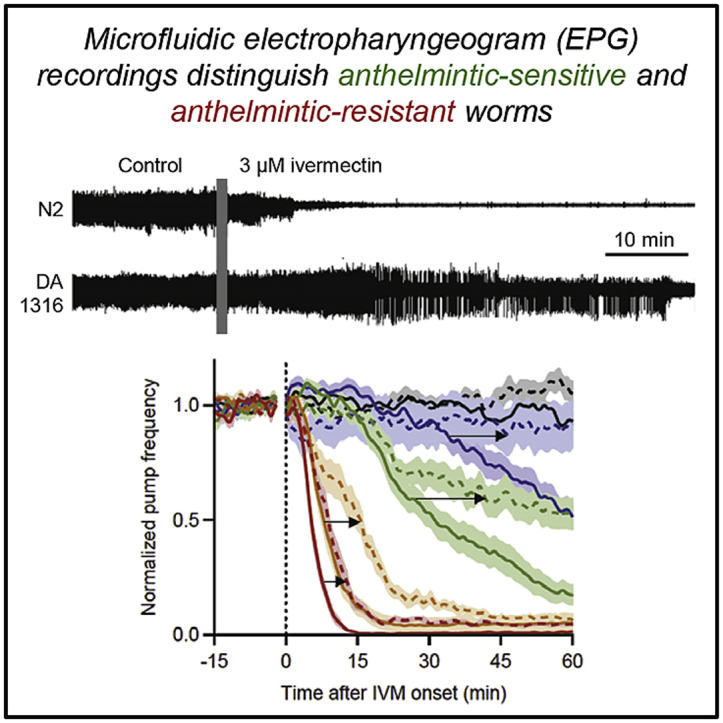

## 1 Introduction

Parasitic infections cause human disability and death in economically disadvantaged regions of the world, with the burden of helminth (parasitic worm) disease exceeded only by malaria (GBD, 2016 Disease and Injury Incidence and Prevalence Collaborators, 2017). Poverty drives these diseases and they in turn trap communities and nations in poverty and ill health. Parasites also impact the health of wildlife, companion animals and agricultural animals, causing economic losses and serving as reservoirs for zoonotic infections of humans (e.g., Otranto et al., 2017; Rashid et al., 2018). The control and elimination of helminth infections benefit from non-pharmacological interventions such as improved animal husbandry as well as sanitary and economic improvements in human communities (Hawdon, 2014). Anthelmintic (anti-parasitic worm) drugs have played a paramount role in combatting these infections, but existing drugs have significant limitations including a limited spectrum of activity across worm species and increases in acquired resistance (e.g., Wolstenholme et al., 2015; Moser et al., 2017). Thus, new anthelmintic therapies, whether from newly discovered drugs or improved versions or combinations of existing drugs, are urgently needed.

Many anthelmintic drugs target ion channels (ICs) and neurotransmitter receptors (NTRs), which generate the electrical activity of neurons and muscles (Wolstenholme, 2011). Perturbation of IC/NTR function disrupts worm behavior, leading to paralysis, starvation, expulsion and/or immune attack (Wolstenholme et al., 2016b). Classes of nematode ICs/NTRs, and representative anthelmintic drugs that target them, include the glutamate-gated chloride channel (GluCl; ivermectin), several subtypes of acetylcholine receptors (AChRs; levamisole), γ-aminobutyric acid receptors (GABA-Rs; piperazine) and the SLO-1 potassium channel (emodepside) (Holden-Dye and Walker, 2014). Nematode IC/NTRs have mammalian orthologs but can be selectively targeted by anthelmintic drugs because of genetic divergence over evolutionary time.

Laboratory assays for detecting anthelmintic activity include worm growth, locomotion/motility, fecundity, and survival, sometimes achieved with high throughput via automation (e.g., Buckingham et al., 2014; Bulman et al., 2015; Burns et al., 2015; Mathew et al., 2016; Partridge et al., 2017). Electrical signals from nematode ICs/NTRs are detectable by a variety of electrophysiological methods (Schafer, 2006) but electrical recordings are not commonly included in drug development pipelines due to technical challenges and relatively low throughput. Electrophysiology is uniquely capable of revealing how anthelmintic drugs and candidate molecules perturb IC/NTR function, for example in examining effects of resistance-conferring genetic mutations and informing SAR (structure-activity relationship) analyses. To facilitate the use of electrophysiology in anthelmintic drug research, we developed a medium-throughput microfluidic platform for recording electropharyngeograms (EPGs)—the electrical signals emitted by muscles and neurons of the pharynx during pharyngeal pumping (feeding)—in *C. elegans* and parasitic nematodes (Lockery et al., 2012; Weeks et al., 2016b). Another microfluidic device for recording EPGs was developed by Holden-Dye and colleagues (Hu et al., 2013; see Section 4). Microfluidic devices (chips) are an increasingly common tool in worm research (reviewed by San-Miguel and Lu, 2013; Muthaiyan Shanmugam and Subhra Santra, 2016) including the study of anthelmintic drugs and drug resistance (Carr et al., 2011; Chen et al., 2011; Liu et al., 2012, 2013; Lycke et al., 2013; Aubry and Lu, 2017; Ding et al., 2017).

Most nematodes have an elongated, muscular pharynx that contracts rhythmically (“pumps’) to pull food (e.g., blood or host tissue in parasitic species, and bacteria in many free-living worms such as *C. elegans*) into the mouth and intestinal tract. In contrast, some species have minimal pharyngeal musculature and may feed by other means (Hüttemann et al., 2007). Pharyngeal muscles and neurons, and the role of ICs/NTRs in their function, are best understood in *C. elegans* (Franks et al., 2006; Avery and You, 2012; Dallière et al., 2017). The *C. elegans* pharynx contains 20 muscle cells, 20 neurons and 3 other cell types, with only a single neuron connecting the pharynx to the rest of the nervous system. Each pump is generated by the contraction, then relaxation, of the corpus, anterior isthmus and terminal bulb regions of the pharynx, sometimes followed by isthmus peristalsis to move ingested bacteria posteriorly. The pumping rhythm is intrinsically myogenic, paced by neural innervation and influenced by neurohormonal modulators (as in the mammalian heart). Likewise, the electrode configuration used to noninvasively record EPGs from intact worms is analogous to that used for a human electrocardiogram (Raizen and Avery, 1994). Pharyngeal muscles are innervated by excitatory cholinergic motoneurons that initiate muscle contraction, and inhibitory motoneurons that release glutamate to terminate each pump. Feeding is modulated by 5-hydroxytryptamine (5HT, serotonin; Song and Avery, 2012; see Section 3.2) and various neuropeptides (Li and Kim, 2008; Holden-Dye and Walker, 2013).

The physiology and life cycle of *C. elegans* differ from those of parasitic nematodes, but *C. elegans* has much to offer anthelmintic research, especially as a highly tractable molecular-genetic model (Holden-Dye and Walker, 2014; Burns et al., 2015; Geary et al., 2015). Importantly, the EPG chip used in the present study has been validated with two major soil-transmitted helminths: the hookworm, *Ancylostoma ceylanicum,* and ascarid worm, *Ascaris suum* (Weeks et al., 2016b). Progress has also been made with *Haemonchus contortus*, an intestinal parasite of sheep and goats (Wolstenholme et al., 2016a), and other nematode species (Weeks, 2016). The present study had two main objectives: (1) to optimize methods for stimulating robust, sustained pharyngeal pumping in *C. elegans* in microfluidic EPG chips and (2) to further validate the use of EPG recordings and automated data analysis by characterizing the effects of three anthelmintic drugs that act on ICs/NTRs to disrupt electrical signaling. The results provide a useful framework for investigators who would like to incorporate electrophysiology into their anthelmintic research workflow.

## 2 Materials and methods

### 2.1 Nematodes

*C. elegans* strains from the *Caenorhabditis* Genetics Center (CGC; Minneapolis, MN) were grown at room temperature using standard methods on Nematode Growth Medium (NGM) agar plates seeded with the OP50 strain of *E. coli*. Control worms (reference strain) were Bristol N2; other strains were DA1316 [*avr-14(ad1305); avr-15(vu227); glc-1(pk54)*; ivermectin resistant]; VC2937 [*unc-38(ok2896)*; levamisole resistant]; CB407 [*unc-49(e407)*; piperazine resistant]; and CB6147 [*bus-8(e2882);* drug hypersensitivity]. Synchronous cultures of worms were obtained by bleaching adults to obtain cohorts of eggs (Stiernagle, 2006). Day-1 adult hermaphrodites (12–24 h after the L4 to adult molt) were used for all experiments.

### 2.2 Device fabrication

Microfluidic chips with 8 recording modules were fabricated using standard soft lithographic methods as described in Lockery et al. (2012). Briefly, silicon wafer masters were created using SU-8 2050 resist (Microchem, Newton, MA) and replica-molded in polydimethylsiloxane (PDMS; Dow Corning Sylgard 184, Corning, NY). Ports, inlets, and fluid reservoirs were punched manually in PDMS castings, which were then bonded to glass substrates after exposure to an oxidizing air plasma. The microfluidic chip design was identical to that in Lockery et al. (2012) with the exception of one improvement (see Section 3.1).

### 2.3 Solutions and drugs

All EPG recordings were made in M9 buffer (Stiernagle, 2006), to which drugs, solvents or bacteria were added. Stocks of 5-Hydroxytryptamine creatinine sulfate complex (5HT, Sigma-Aldrich H7752; St. Louis, MO) were prepared in M9 at 40 mM, stored at −20 °C and diluted to desired concentrations in M9. Chip perfusion was initiated within 70 min of preparing a 5HT solution. Ivermectin (IVM; Sigma-Aldrich I8898) stocks (5 mM) were prepared in 100% DMSO and stored at −20 °C. The highest concentrations of DMSO that did not perturb EPG activity in control experiments were 0.2% (Fisher D-136) or 0.5% (Sigma-Aldrich Hybri-Max D2650) (data not shown) so these were the highest concentrations used in working solutions. Levamisole hydrochloride (LEV; Sigma 31,742) stocks (100 mM) were prepared in dH_2_0 and stored at 4 °C until dilution on the day of use. Piperazine hexahydrate (PPZ; Sigma P7003) stocks (1M) were prepared in dH_2_0 and stored at 4 °C until use. The pH of PPZ solutions was adjusted to 7.0 using HEPES buffer in M9 and titrating with 12 N HCl. Working solutions of all anthelmintic drugs were prepared from stocks and used on the same day. In some initial experiments, 0.005% Fast Green (Fisher F-99) was added to solutions to confirm uninterrupted perfusate flow (Lockery et al., 2012).

### 2.4 Preparation of *E. coli* OP50

Standard methods (Stiernagle, 2006) were used to grow liquid cultures of OP50. A small volume of an existing culture in Luria-Bertani (LB) medium was inoculated into a 200 ml flask of fresh LB and rocked overnight at 180 rpm at 37 °C. Fifty ml of the culture was then centrifuged for 8 min at 5000 rpm and the pellet rinsed in dH_2_0. This procedure was repeated and the pellet was resuspended in 10 ml M9. The optical density at 600 nm (O.D._600_; Stevenson et al., 2016) was measured spectrophotometrically (LAXCO DSM Density Meter, Bothell, WA) after diluting the suspension with M9 (typically 1:10) to lie within the linear range of the spectrophotometer. Two independent dilutions were tested and averaged to obtain the O.D._600_ value. The stock suspension was then diluted in M9 to obtain the desired working concentration, which was confirmed spectrophotometrically (mean of 2 independent readings). The stock OP50 suspension in M9 was stored at 4 °C, used for no more than 5 d, and O.D._600_ was determined before each use. As needed, new aliquots were collected from the original LB culture flask, which was kept at 4 °C and discarded after 30 d. In some experiments (see Section 3.3), O.D._600_ readings were made both before and after OP50 suspensions were perfused through chips.

### 2.5 Loading and recording worms

**Fig. 1:**
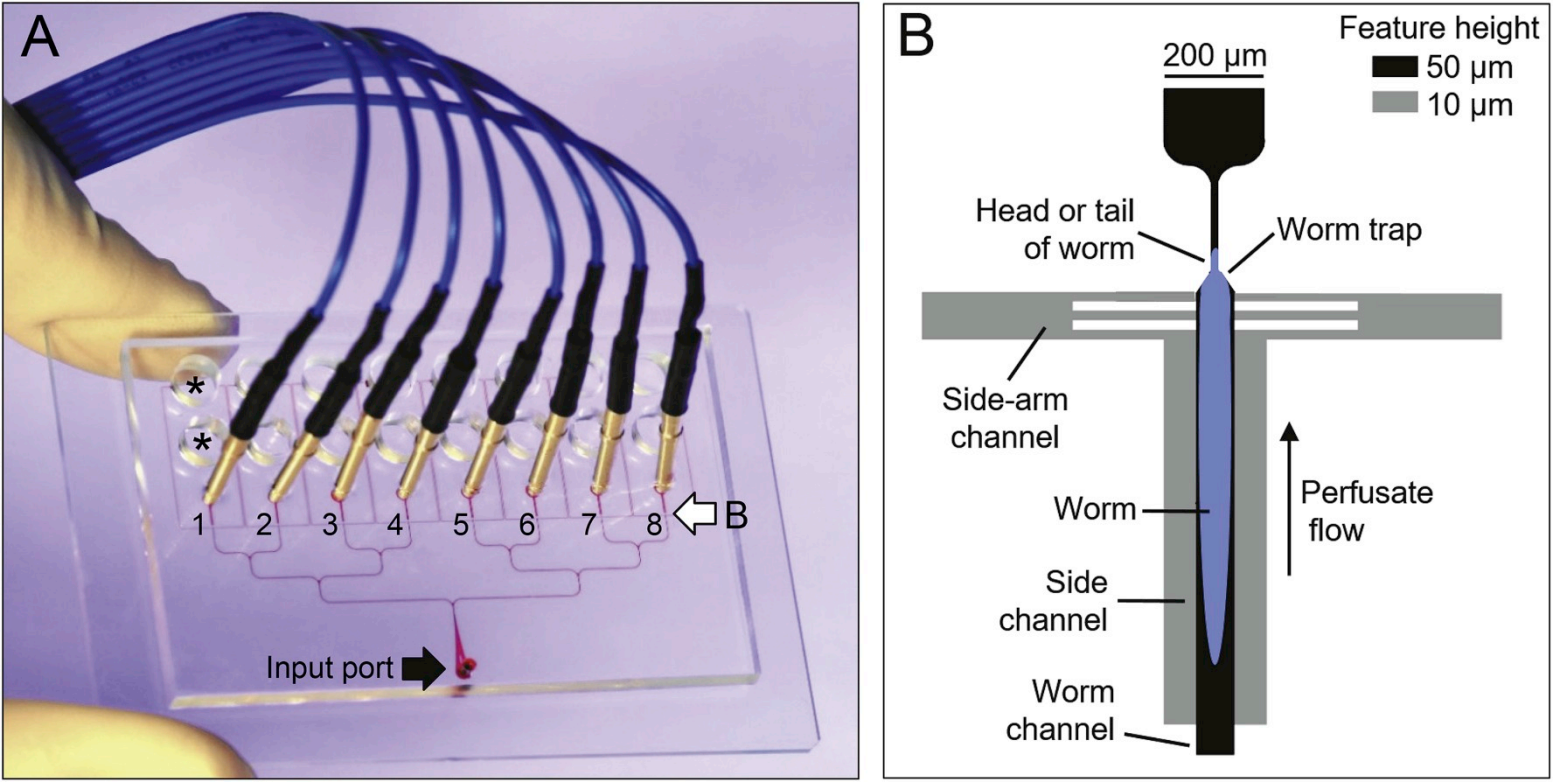
Microfluidic EPG recording device (chip). **A.** In this image (modified from Weeks et al., 2016b; https://creativecommons.org/licenses/by/4.0/), microchannels were filled with a red dye to aid visualization. *C. elegans* were loaded into the input port (filled arrow) and distributed via a branching network of channels into 8 recording modules, each with a distal (blue) electrode wire. A hollow metal electrode (not shown) inserted into the input port delivered perfusate and served as a common electrical reference. Solutions flowed past worms and exited to waste reservoirs (*, waste reservoirs for recording module 1). Open arrow indicates expanded region shown in **B**. **B.** Recording modules were modified from an earlier design by adding bilateral side channels parallel to the worm channel to enhance access of perfused solutions to worms. Feature height in the PDMS layer of the chip was measured relative to the glass substrate. (For interpretation of the references to color in this figure legend, the reader is referred to the Web version of this article.)

Methods were mostly the same as in Lockery et al., (2012), Weeks et al. (2016b), and Weeks et al. (2018). In brief, Day-1 adults were transferred from the OP50 lawn on NGM plates into a glass well containing M9 to acclimate for 10 min. In many experiments, treatment with 10 mM 5HT in M9 (termed “M9-5HT”) was begun at this time. For some experiments (see Results and Discussion) worms were transferred from OP50 plates to unseeded NGM plates for a period of food deprivation before experiments. Chips were loaded under a stereomicroscope by placing 8 worms in the input port of a chip (see Fig. 1A) and using tubing and a syringe loaded with M9 or M9-5HT to gently propel them into the worm trap within each recording module (see Fig. 1B). Worms lodged either head-first or tail-first in the recording modules and, by convention (Raizen and Avery, 1994), EPG traces in the Figures are displayed with the E spike (pharyngeal muscle excitation) of the pump waveform upward and the R spike (pharyngeal muscle relaxation) downward. After loading, a recording electrode was inserted distal to each recording module. A hollow reference electrode was inserted in the input port and connected via tubing to a syringe controlled by a syringe pump (Harvard Apparatus PHD, 2000; Holliston, MA) running at 6 μl/min. To change solutions, the reference electrode was removed and replaced with an electrode leading to a syringe containing the new perfusate. The new perfusate reached worms within 60 s of switching the perfusion source. The brief electrical artifact produced by switching perfusion lines was blanked in the Figures. After passing over worms, perfused solutions accumulated in on-chip waste reservoirs.

EPGs were recorded as in Lockery et al. (2012) and Weeks et al. (2018) with few modifications. Electrodes were led to a head-stage signal conditioning board that contained a separate unity-gain voltage follower for each worm channel (fabricated in-house) and then to AC differential amplifiers (A-M Systems model 1700, Carlsborg, WA) for amplification (1000x) and band-pass filtering (1–1000 Hz, plus a 60 Hz notch filter). These signals were sent to a data acquisition system [Micro1401-3, Cambridge Electronic Design (CED), Cambridge, UK] and Spike2 software (version 7.06a, CED) at 2.5 KHz per channel.

*bus-8* worms, which have disrupted epidermis and altered surface properties (Partridge et al., 2008), tended to adhere to PDMS during chip loading. This problem was reduced by pre-treating degassed chips (10 min degassing in a vacuum chamber) with Pluronic F-127 (Sigma-P2443-250G; Wu and Hjort, 2009; Krajniak and Lu, 2010) to reduce stickiness. Pluronic F-127 was prepared at 5% in dH_2_O heated to 55 °C. Before use, the solution was warmed to 65 °C, injected into a chip's microfluidic channels via syringe, and incubated for 3 min. The channels were then rinsed twice (65 °C M9 and room temperature M9) and loaded as above. Chips pre-treated with Pluronic F-127 were used for both *bus-8* and N2 control worms.

### 2.6 EPG data analysis

We found no significant power in EPG signals related to pharyngeal pumping at frequencies above 100 Hz. Therefore, except when measuring high-frequency voltage noise associate with the seal resistance (*R*_*seal*_; see Section 2.7), Spike2 data were down-sampled to 500 Hz/channel before exporting to Igor Pro (WaveMetrics, Lake Oswego, OR, USA). They were further down-sampled to 250 Hz in Igor Pro, before analysis using a pump-detection algorithm described briefly in past publications (Weeks et al., 2016b, 2018). A complete description of the method follows. We previously documented strong concordance between pumps identified by the algorithm and blinded human observers (Weeks et al., 2016b).

In the canonical EPG waveform (Raizen and Avery, 1994) recorded from worms positioned head-first in a pipette, the E and R spikes have the greatest amplitudes, making pumps potentially identifiable by threshold detection alone. However, in many recordings (including after drug treatment), pump waveforms were more complex and required additional pre-processing to increase the amplitude of the rapid E and R spikes relative to slower components and background noise, and to compensate for variations in EPG amplitude during a recording. Recordings from worms positioned tail-first in recording modules were inverted to direct the E spikes upward prior to data analysis. The automated algorithm used a random search strategy to find the combination of 6 parameter values that gave the most reliable pump detection, as evidenced by maximizing a fitness function.

Automatic gain control was essential because slow variations in EPG amplitude during the course of a 90-min recording were often so large that a uniform threshold could not be used without some kind of amplitude normalization. Our automatic gain control consisted of dividing the voltage time series by a low-pass filtered version of itself (Gaussian filter, σ = 500 ms, applied to the absolute value of the EPG data), to give a normalized measure of EPG amplitude. The normalized signal was passed to separate detectors for E spikes and R spikes. Each of the two detectors consisted of a linear bandpass filter followed by a detector of peaks exceeding a threshold, and had 3 adjustable parameters: low frequency cut-off ($f_{low}\geq 1\;\text{Hz}$), high frequency cut-off ($f_{high}\leq 125\;\text{Hz}$), and threshold. The resulting 6 parameters were optimized for each worm, using a random search algorithm to find the combination of parameter values that maximized the fitness function.

The fitness function was based on the human observation that the EPG waveform corresponding to a pump consists of an E spike followed with fairly constant delay by an R spike. We therefore required that each pump began with a detected E spike and ended with a detected R spike, and used the distribution of time intervals from each E spike to the next R spike (i.e., the pump duration) as the basis for the optimization procedure. Unpaired E spikes and R spikes were counted as “orphans.” Informally stated, the optimization procedure sought to adjust the thresholds and filter frequencies such that detected E spikes were almost always followed by detected R spikes, with few orphans and a narrow distribution of pump durations. We used an entropy measure of distribution width,

$$W=\sum_{d_{min}}^{d_{max}}\frac{d_{cum}\cdot log_{2}\left(d_{cum}\right)-p_{cum}\cdot log_{2}\left(p_{cum}\right)}{d_{mean}-d_{min}},$$

where $d_{min}=30\;\text{ms}$ and $d_{max}=500\;\text{ms}\;$ are the minimum and maximum allowable pump durations, respectively, $d_{mean}$ is the mean pump duration, $d_{cum}\left(t\right)$ is the observed cumulative distribution of pump durations,

$$d_{cum}\left(t\right)=\frac{\#\;of\;pump\;durations\leq t}{total\;\#\;of\;pump\;durations},$$

and $p_{cum}\left(t\right)$ is the theoretical cumulative distribution of pump durations under the null hypothesis that R spikes occur at random times (Poisson process) having the same mean pump duration and constrained by $d_{max}$ and $d_{min}$

$$p_{cum}\left(t\right)=\frac{1-e^{-(t-d_{min})/(d_{mean}-d_{min})}}{1-e^{-(t-d_{max})/(d_{mean}-d_{min})}}.$$

W is positive if the observed distribution of pump durations is narrower than a random distribution.

The fitness function to be maximized was

F=PPS·W−OPS·0.01,

where PPS was the mean frequency of detected pumps, and OPS was the mean frequency of orphan E spikes and R spikes. F was maximized using a random search algorithm similar to that described in Roberts et al. (2016). The output of the pump-detection algorithm was a list of all detected pumps, in which each pump had a start time (E spike), end time (R spike) and an amplitude for the E and R spikes, from which pump frequency, pump amplitude, inter-pump intervals (IPIs) and other parameters were extracted. Amplitudes were measured from EPG recordings at the 250 Hz sample rate, with no normalization or filtering except for a 60 Hz notch filter.

**Fig. 2:**
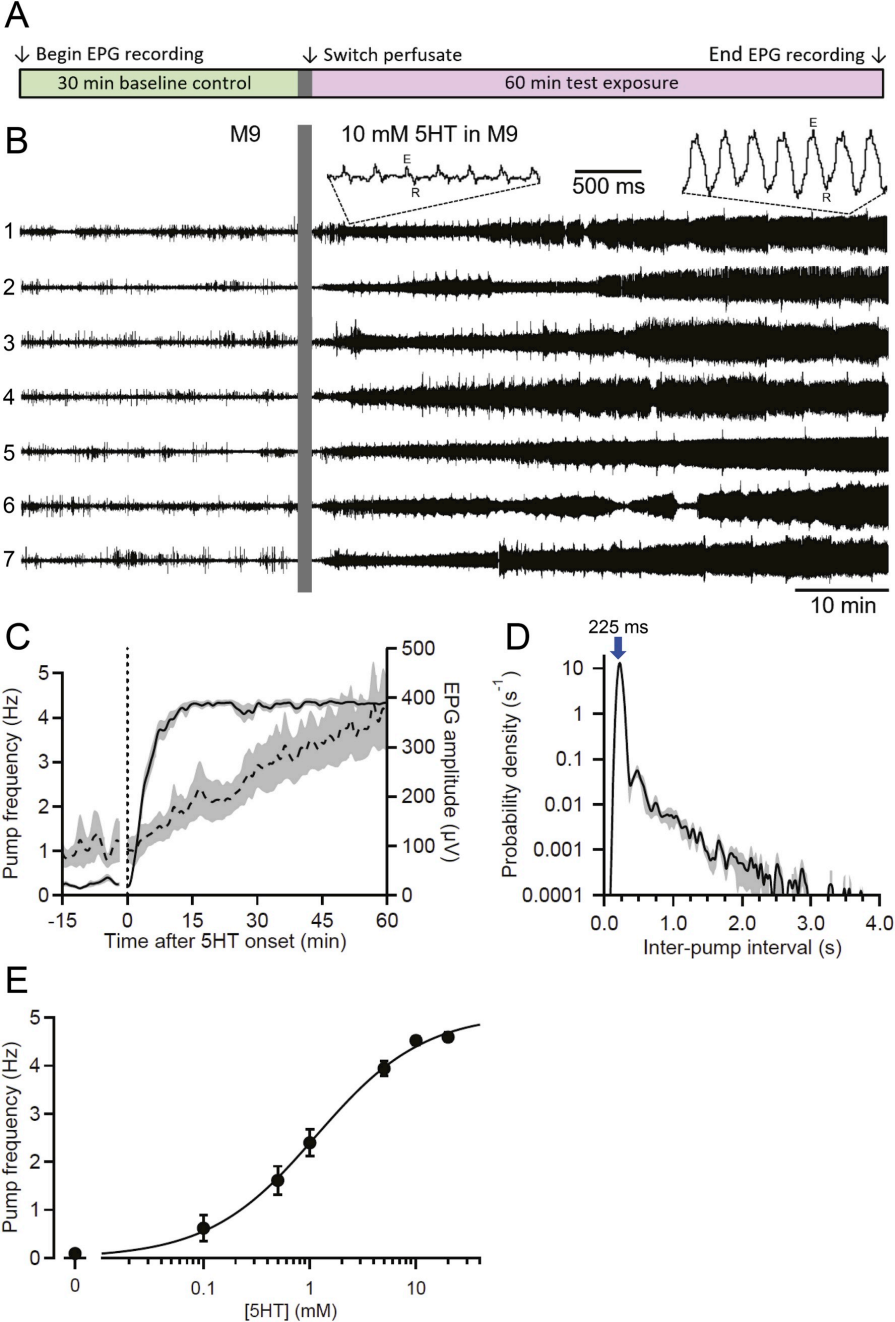
Stimulation of pharyngeal pumping by 5HT. **A**. Experimental protocol for most experiments. Worms were perfused with a control solution for 30 min, followed by switching the perfusate to a test solution and recording for 60 min more. **B.** Representative example of 5HT-stimulated EPG activity. Simultaneous EPG recordings from seven N2 worms (numbered 1 to 7) in one chip: each trace from a different worm. Baseline pumping in M9 was followed by switching (vertical bar masks electrical artifact) to 10 mM 5HT in M9. Pumping increased rapidly and EPG amplitude increased over time (insets show pumps from worm 1; E, excitatory spike, R, relaxation spike). Worms 1, 5 and 6 were head-first in worm traps while the others were tail-first. **C.** Effect of 5HT on pump frequency and amplitude. Switch to 10 mM 5HT occurred at the vertical dotted line, with the electrical artifact blanked. Time-series data were extracted using custom software (see Section 2.6) and plotted as mean pump frequency (solid line) and amplitude (dashed line; peak-to-peak from E spike to R spike) (shading depicts S.E.M. in all Figures; *n* = 19 worms). **D**. IPI probability density histogram for worms in 10 mM 5HT, during *t* = 30–60 min post-switch (same worms as **C**). The mode of the distribution is marked (arrow; 225 ms, or 4.44 Hz). **E**. Steady-state pump frequency increased with 5HT concentration. Worms were perfused for 60 min with 5HT to achieve steady-state pump frequency, which was measured for the next 30 min (*t* = 60–90 min). Plot shows mean ± S.E.M. (*n* = 19–23 worms/group) pump frequency for different 5HT concentrations, fitted using the Hill equation (maximum frequency, 5.08 Hz; C_1/2_, 1.15 mM; Hill coefficient, 0.86). Some error bars are smaller than the symbols.

**Fig. 3:**
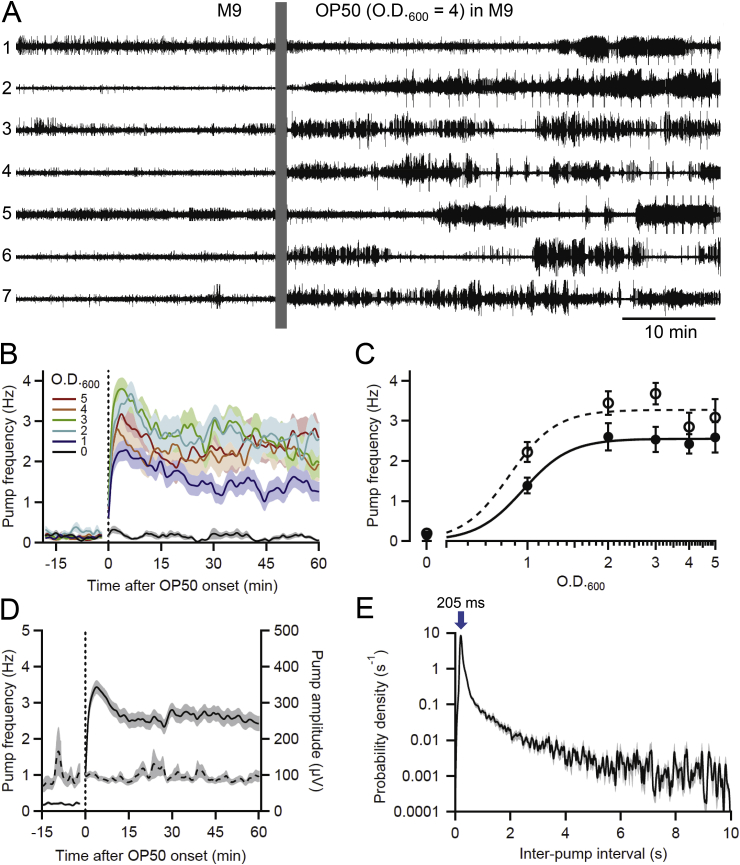
Stimulation of pharyngeal pumping by *E. coli* OP50. **A.** Representative example of simultaneous EPG recordings from seven N2 worms (numbered 1 to 7) in one chip; each trace from a different worm. Baseline activity recorded in M9 was followed by switching (vertical bar masks electrical artifact) to OP50 (O.D._600_ = 4) in M9; the switch marked the termination of a 2-h period of food deprivation. Pumping increased and occurred in irregular bouts. Worms 1 and 5 were head-first in worm traps while the others were tail-first. **B.** Time course of pumping stimulation by OP50. Perfusate switch occurred at the vertical dotted line, with the electrical artifact blanked. Lines show mean pump frequency in worms perfused with OP50 at O.D._600_ values between 0 (blank control) to 5 (*n* = 19–33 worms/group). Concentrations are denoted by color (see key). **C.** Concentration-response curves for rapid and sustained responses to OP50. Plots show mean pump frequency measured from *t* = 2–7 min (open circles and dashed line; rapid response) or *t* = 30–60 min (filled circles and solid line; sustained response) following the switch to OP50. The lowest OP50 concentration tested (O.D._600_ = 1) evoked nearly half-maximal pump frequencies, which implies that C_1/2_ ≅ 1 mM for both rapid and sustained responses, but more data would be required to accurately constrain C_1/2_ and the Hill coefficient. The smooth curves in Fig. 3C, which are best fits to the Hill equation calculated assuming a Hill coefficient = 5, show saturating values of 3.37 Hz and 2.55 Hz for rapid and sustained responses, respectively. **D**. Effect of OP50 on pump frequency and amplitude. Data from worms perfused with OP50 between O.D._600_ = 2–5 were combined and plotted as mean pump frequency (solid line) and peak-to-peak amplitude (dashed line; *n* = 90 worms). Switch to OP50 occurred at the vertical dotted line, with the electrical artifact blanked. **E**. IPI probability density histogram for worms perfused with OP50 between O.D._600_ = 2–5, from *t* = 30–60 min (*n* = 90 worms). The mode of the distribution is marked (arrow; 205 ms, or 4.89 Hz). Plots in panels **B-E** are from the same groups of worms. Some error bars are smaller than the symbols. (For interpretation of the references to color in this figure legend, the reader is referred to the Web version of this article.)

**Fig. 4:**
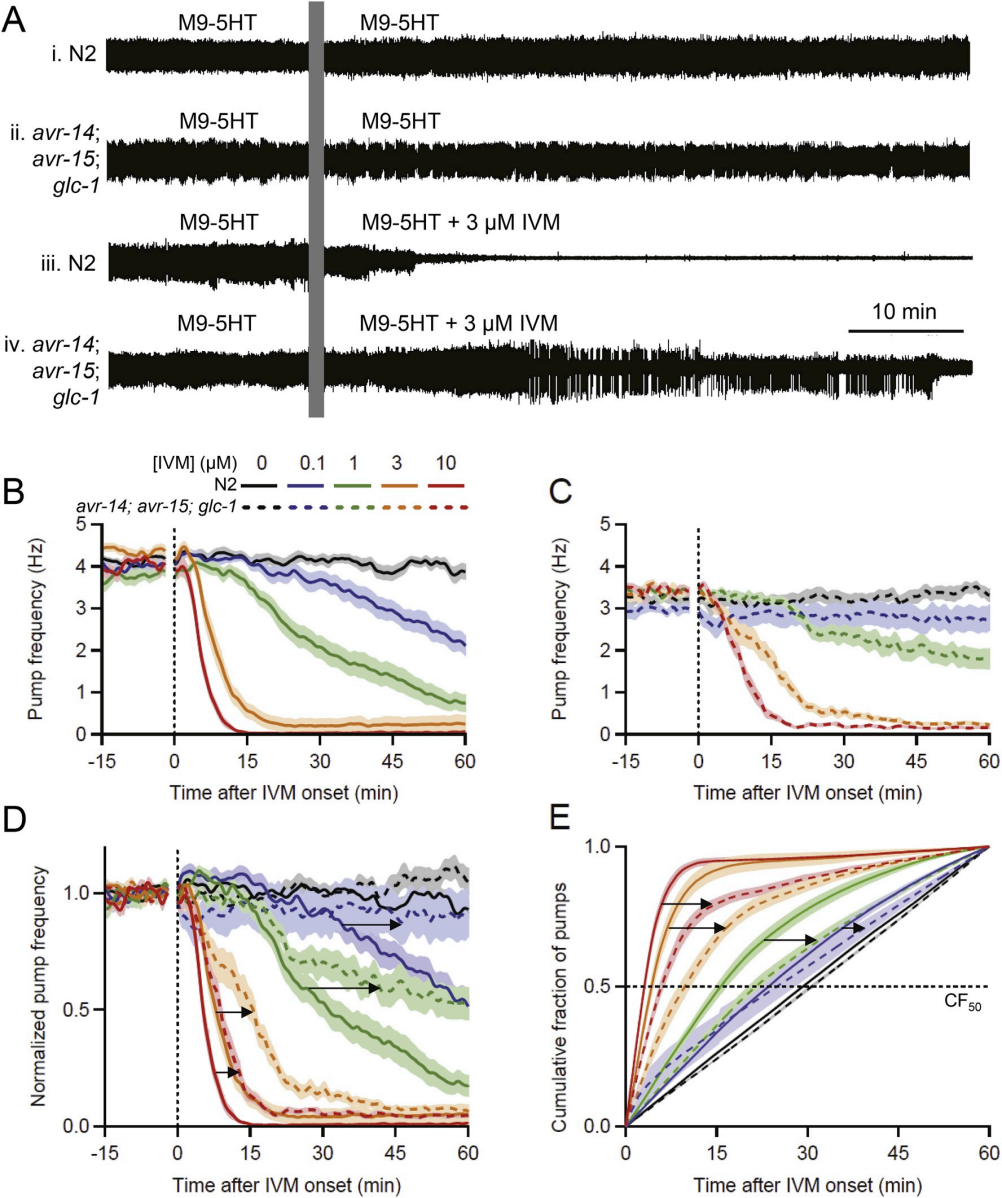
*avr-14*; *avr-15*; *glc-1* (IVM-resistant) worms are less sensitive to IVM than N2s. **A.** Representative EPG recordings from individual worms, all perfused with M9-5HT during the baseline period. Vertical bar masks electrical artifact when the perfusate was switched. **i**, **ii**, N2 and *avr-14*; *avr-15*; *glc-1* worms switched to M9-5HT (controls); **iii**, **iv**, N2 and *avr-14*; *avr-15*; *glc-1* worms switched to 3 μM IVM in M9-5HT. IVM inhibited pumping more strongly in N2 than *avr-14*; *avr-15*; *glc-1*. **B, C.** Pump frequency plotted against time for N2 (**B**) and *avr-14*; *avr-15*; *glc-1* (**C**) worms switched at *t* = 0 min to different concentrations of IVM (mean ± S.E.M.; *n* = 14–35 worms/group). The key in **B** applies to all panels; IVM concentration is denoted by color and genotype by line type (solid, N2; dashed, *avr-14*; *avr-15*; *glc-1*). Perfusate switch occurred at the vertical dotted line, with the electrical artifact blanked. IVM caused concentration-dependent inhibition of pumping in both strains. **D.** Same data as in **B** and **C**, displayed together after normalizing pump frequency to its mean value between *t* = −12 to −2 min within each worm, to correct for different baseline frequencies (see Section 2.6). Pump frequency plots were right-shifted (arrows) in *avr-14*; *avr-15*; *glc-1* relative to N2 worms at each IVM concentration, indicating resistance. **E**. Same data, plotted as the cumulative fraction (CF) of pumps occurring over time after perfusate switch at *t* = 0 min, for each strain and IVM concentration. Dotted line denotes CF_50_, the intercept at which statistical comparisons were made. CF_50_ median values were (N2, *avr-14*; *avr-15*; *glc-1*, respectively, in minutes): 0 μM IVM, 30.8, 29.5; 0.1 μM IVM, 29.6, 26.0; 1 μM IVM, 15.0, 21.2; 3 μM IVM, 4.3, 10.9; and 10 μM IVM, 3.0, 6.0. Arrows denote rightward shifts of CF plots in *avr-14*; *avr-15*; *glc-1* vs. N2 worms. (For interpretation of the references to color in this figure legend, the reader is referred to the Web version of this article.)

**Fig. 5:**
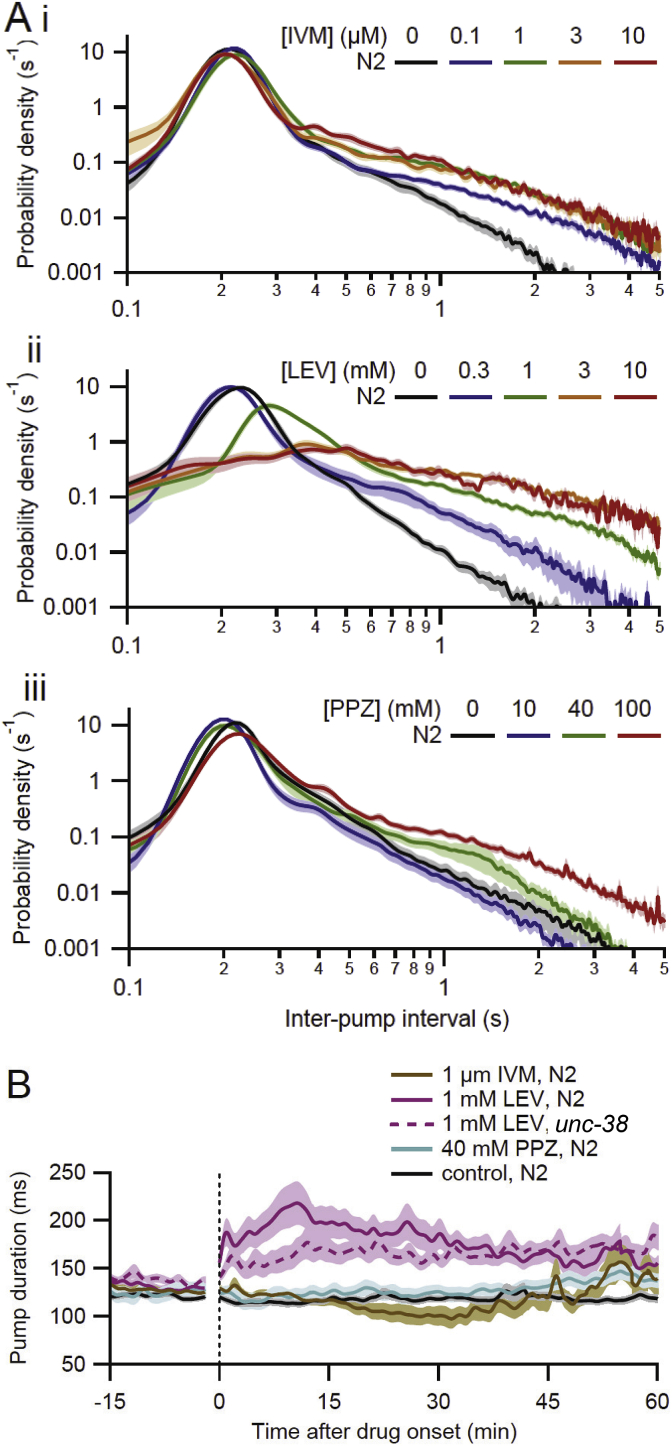
Inter-pump interval (IPI) and pump duration data for three anthelmintic drugs. **A**. IPI probability density histograms from N2 worms during *t* = 0–60 min for **i.** IVM, **ii.** LEV and **iii.** PPZ (same data as Fig. 4, Fig. 7, Fig. 9, respectively). Drug concentrations are given in keys. Control data are from Fig. 4. **B.** Plot of pump duration (interval from E to R spike) during *t* = −15 to 60 min for same three drugs; see key. Perfusate switch occurred at the vertical dotted line, with the electrical artifact blanked. Data are shown for a representative, intermediate concentration of each drug at which pumping continued for the entire 60-min post-switch period. All data are from N2 worms (solid lines) except for *unc-38 (*LEV-resistant; dashed line), included for comparison. (For interpretation of the references to color in this figure legend, the reader is referred to the Web version of this article.)

**Fig. 6:**
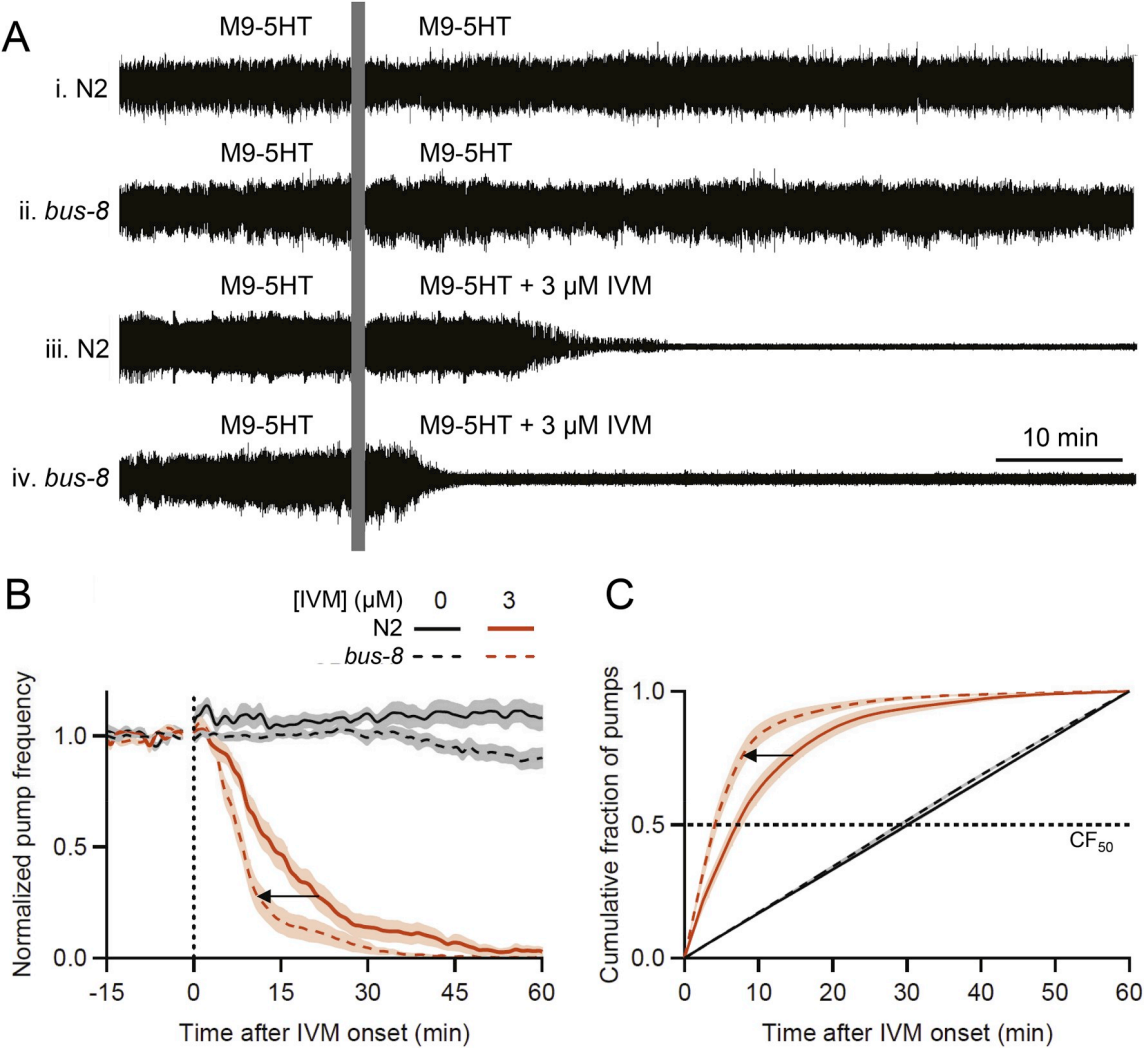
*bus-8* (disrupted cuticle and epidermis) worms are more sensitive to IVM than N2s. Chips used for both strains were pre-treated with Pluronic F-127 to facilitate loading *bus-8* worms, which adhere to PDMS (see Section 2.5). **A.** Representative EPG recordings from individual worms, all perfused with M9-5HT during the baseline period. **i**, **ii**, N2 and *bus-8* worms switched to M9-5HT (controls); **iii**, **iv**, N2 and *bus-8* worms switched to 3 μM IVM in M9-5HT. IVM inhibited pumping more rapidly in *bus-8* than in N2 worms. **B**. Normalized pump frequency (see Section 2.6) plotted against time for N2 and *bus-8* worms switched to 3 μM IVM (mean ± S.E.M.; *n* = 18–29 worms/group). The key in **B** applies to **B** and **C**. Arrow denotes leftward shift of pump frequency plot for *bus-8* compared to N2 worms, indicating increased sensitivity to IVM. **C**. Same data as **B**, plotted as the cumulative fraction (CF) of pumps occurring over time after perfusate switch at *t* = 0 min. Dotted line denotes CF_50_, the intercept at which statistical comparisons were made. CF_50_ median values were (N2, *bus-8*, respectively, in minutes): 0 μM IVM, 30.1, 29.3; 3 μM IVM, 7.7, 4.2. Arrow denotes leftward shift of CF plot in *bus-8* relative to N2s.

**Fig. 7:**
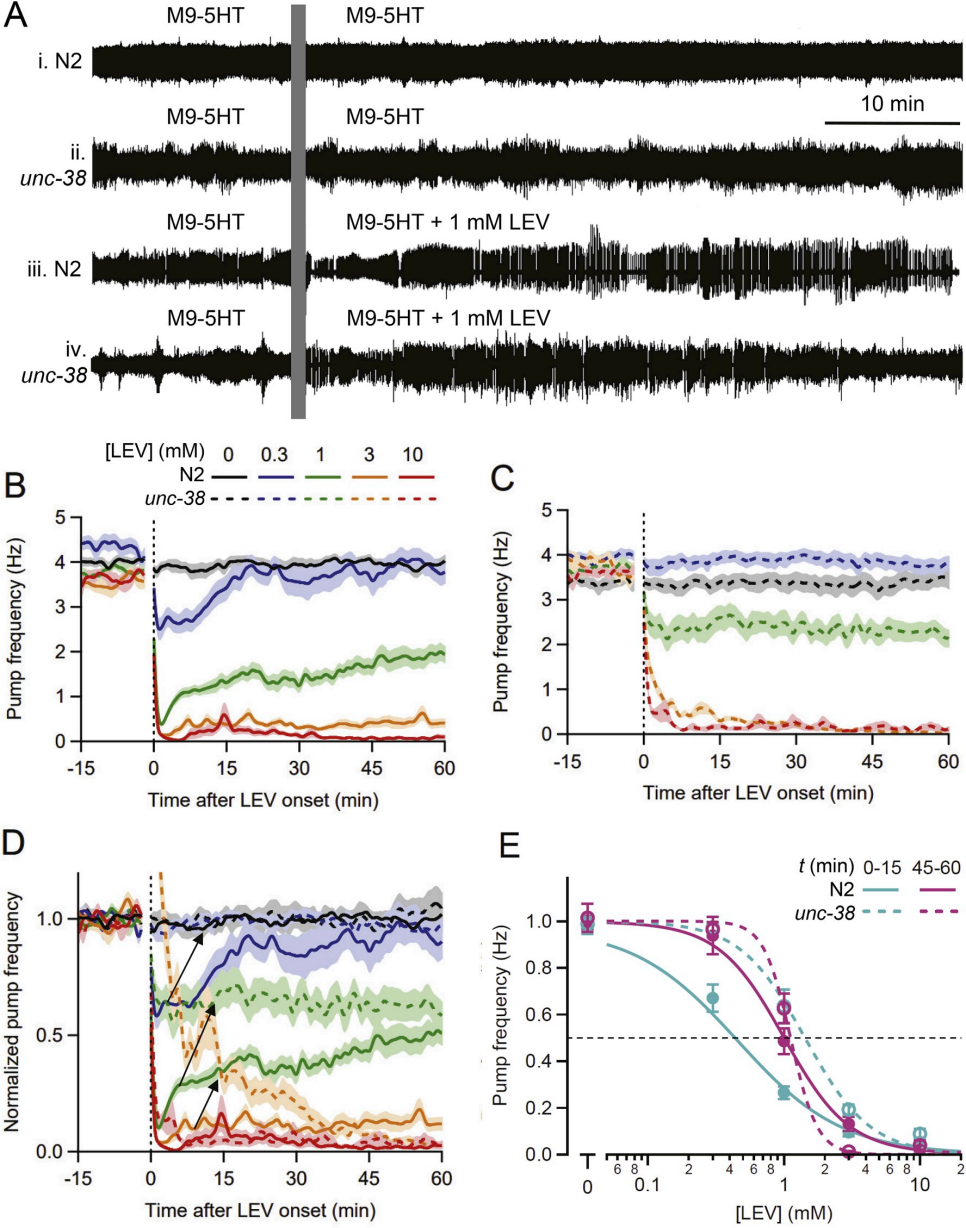
*unc-38* (LEV-resistant) worms are less sensitive to LEV than N2s. **A.** Representative EPG recordings from individual worms, all perfused with M9-5HT during the baseline period. **i**, **ii**, N2 and *unc-38* worms switched to M9-5HT (controls); **iii**, **iv**, N2 and *unc-38* worms switched to 1 mM LEV in M9-5HT. LEV inhibited pumping more strongly in N2 than in *unc-38*. **B, C.** Pump frequency plotted over time for N2 (**B**) and *unc-38* (**C**) worms switched at *t* = 0 min to different concentrations of LEV (mean ± S.E.M., *n* = 16–32 worms/group). In all panels, concentrations are denoted by color and genotype by line type (solid, N2; dashed, *unc-38*). Perfusate switch occurred at the vertical dotted line, with the electrical artifact blanked. LEV caused concentration-dependent inhibition of pumping in both strains. **D.** Same data as in **B** and **C**, displayed together after normalizing pump frequency. N2s showed an early phase of inhibition compared to *unc-38* worms; arrows denote reduced inhibition in *unc-38* compared to N2 worms at 0.3, 1 and 3 mM LEV. **E**. Same data, fitted to the Hill equation for post-switch interval *t* = 0–15 min (“rapid” inhibition; teal lines) and *t* = 45–60 min (“sustained” inhibition; pink lines). Dashed line denotes EC_50_ (LEV concentration at which pump frequency was reduced by half). EC_50_ values (mean ± S.E.M.) for LEV were: N2 rapid, 0.46 ± 0.06 μM; N2 sustained, 1.00 ± 0.12 μM; *unc-38* rapid, 1.38 ± 0.15 μM; *unc-38* sustained, 1.14 ± 0.07 μM. Statistical comparisons are provided in Section 3.6. (For interpretation of the references to color in this figure legend, the reader is referred to the Web version of this article.)

**Fig. 8:**
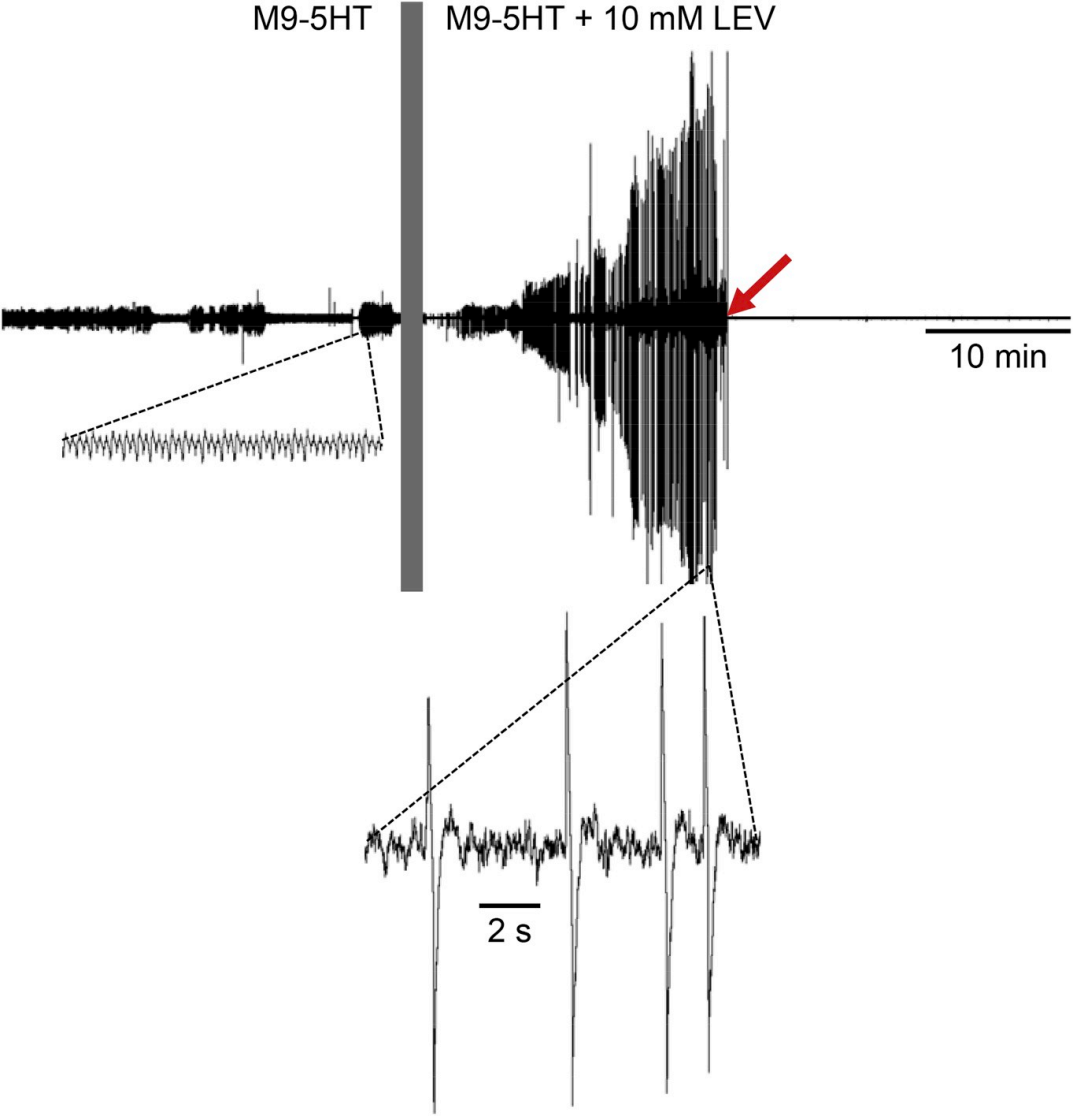
LEV-induced paralysis expels worm through worm trap. Trace shows a representative EPG recording from an N2 worm; vertical bar marks perfusate switch from M9-5HT to 10 mM LEV in M9-5HT. During baseline, the EPG signal was of normal amplitude (inset) (in this example, baseline pumping had gaps). Following the switch to LEV, EPG signal amplitude increased (inset), followed by loss of the signal (red arrow) when the worm was expelled from the chip's recording module into the waste reservoir. All traces are shown at the same vertical scale. (For interpretation of the references to color in this figure legend, the reader is referred to the Web version of this article.)

**Fig. 9:**
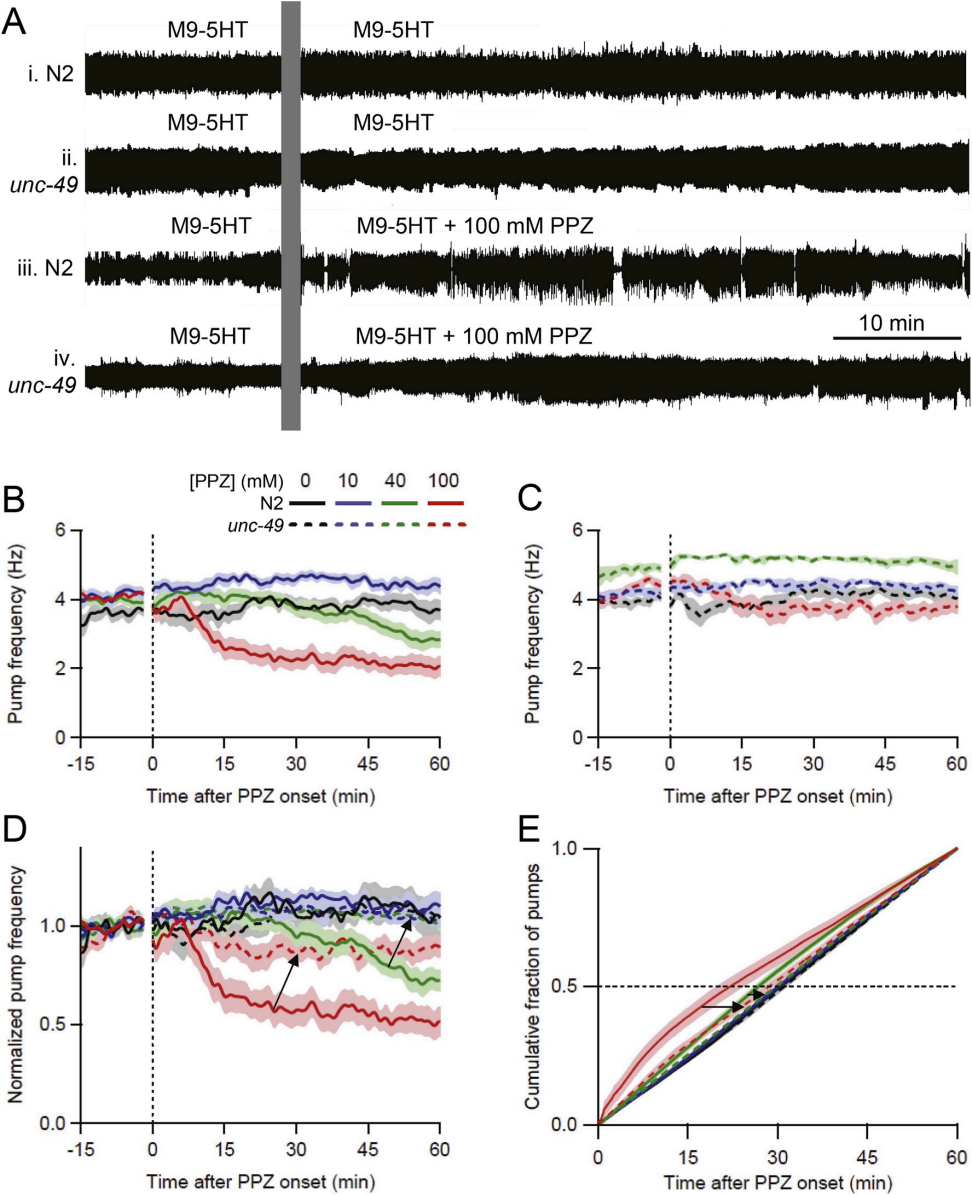
*unc-49* (PPZ-resistant) worms are less sensitive to PPZ than N2s. **A.** Representative EPG recordings from individual worms, all perfused with M9-5HT during the baseline period. **i**, **ii**, N2 and *unc-49* worms switched to M9-5HT (controls); **iii**, **iv**, N2 and *unc-49* worms switched to 100 mM PPZ in M9-5HT. PPZ inhibited pumping more strongly in N2 than *unc-49* worms. **B, C.** Pump frequency plotted over time for N2 (**B**) and *unc-49* (**C**) worms switched at *t* = 0 min to different concentrations of PPZ (mean ± S.E.M., *n* = 17–29 worms/group). In all panels, concentrations are denoted by color and genotype by line type (solid, N2; dashed, *unc-49*). Perfusate switch occurred at the vertical dotted line, with the electrical artifact blanked. **D.** Same data as in **B** and **C**, displayed together after normalizing pump frequency. PPZ caused concentration-dependent inhibition of pumping in both strains; arrows denote reduced inhibition in *unc-49* compared to N2 worms at 40 and 100 mM PPZ. **E**. Same data, plotted as the cumulative fraction (CF) of pumps occurring over time after perfusate switch at *t* = 0 min, for each genotype and PPZ concentration. Dotted line denotes CF_50_, the intercept at which statistical comparisons were made between groups. CF_50_ median values were (N2, *unc-49*, respectively, in minutes): 0 mM PPZ, 30.4, 30.7; 10 mM PPZ, 30.1, 30.1; 40 mM PPZ, 28.4, 29.9; and 100 mM PPZ, 22.3, 29.3. Arrows denote same difference between strains as in **D**. (For interpretation of the references to color in this figure legend, the reader is referred to the Web version of this article.)

Some worms were excluded from analysis based upon the inability to detect pumps reliably, or a low baseline pump frequency (“baseline” being the period prior to the onset of a test solution; see Fig. 2A). In all experiments, worms were rejected if pump detection was deemed unreliable, as indicated by W being below a criterion level (Fig. 2, Fig. 3, W<0.1; all other experiments, W<0.25). When administering an anthelmintic drug in the presence of 5HT (see Fig. 4, Fig. 5, Fig. 6, Fig. 7, Fig. 8, Fig. 9), worms were excluded if they had a low baseline pump frequency (<2 Hz mean from *t* = −12 to −2 min, with *t* = 0 min being the onset of a test solution). Overall, 74 of 2134 worms (4.5%) analyzed for Fig. 4, Fig. 5, Fig. 6, Fig. 7, Fig. 8, Fig. 9 were rejected based on a low baseline pump frequency; an additional 95 worms (4.4%) were rejected because W<0.25. More accepting criteria were used for worms analyzed in Fig. 2, Fig. 3, in which no pumping stimulus was applied prior to stimulation by 5HT or OP50. Therefore, none of the 310 worms were rejected due to a low baseline frequency, but 21 worms (6.8%) were rejected because W<0.1.

To compare baseline pump frequencies between N2 and mutant *C. elegans* strains, a mean pump frequency was determined from *t* = −12 to −2 min for each worm and these means were averaged to obtain a baseline pump frequency for each genotype.

### 2.7 Noise analysis of EPG recordings

To infer changes in *R*_*seal*_ between a worm's body and the worm trap (Lockery et al., 2012), EPG recordings in Spike2 acquired at 2.5 kHz were filtered (high pass, 4th order Bessel filter with corner frequency of 200 Hz), and RMS (root mean square) noise amplitude was computed with a 2 ms time constant. Data segments of interest (2000 ms/segment) were decimated to a sample interval of 10 ms and exported as text files to Excel for further analysis. Reported values are from data segments that lacked residual artifacts from EPG waveforms after filtering.

### 2.8 Display and statistical analysis of data

Because of potential variation in *R*_*seal*_ during recordings of a particular worm, and between different worms in their respective recording channels, we do not report EPG signal amplitudes during anthelmintic treatments. EPG traces are displayed in Figures with the vertical gain (voltage) adjusted to give similar peak-to-peak amplitudes during the baseline period. Transient electrical artifacts or occasional large pump signals were masked so that traces could be shown with similar gains.

Plots of pump frequency vs. time were constructed by counting pumps in 1-s time bins and smoothing the values using a Gaussian weighted sliding window (S.D. = 30 s). The smoothed frequency vs. time curves were averaged across all worms in each experimental group (ensemble average; *n* = number of worms in the ensemble) and plotted as mean (line) ± 1 S.E.M. (shading). Baseline pump frequency was obtained by measuring each worm's mean pump frequency for a 10-min interval (*t* = −12 min to −2 min), then averaging across worms. “Normalized” pump frequency was calculated by dividing each worm's smoothed frequency vs. time curve by its mean pump frequency during the baseline period, then averaging the normalized pump frequency data across worms.

To statistically compare the time required for inhibitory drug effects, we computed the cumulative fraction (CF) of pumps that occurred following drug onset, with CF_50_ defined as the time at which 50% of the total number of pumps occurred during the 60 min observation period after onset of a test solution (Weeks et al., 2016b). This cumulative measure provided an unambiguous *CF*_50_ value for each worm, regardless of how irregular its pumping became. Some data were fitted using the Hill equation to derive EC_50_ values (concentration at which pump frequency declined by half), as described previously (Weeks et al., 2018).

Mean values were compared by 1-tailed or 2-tailed non-parametric tests (details in Section 3). EC_50_ values from Hill plots were compared using a likelihood ratio test (Weeks et al., 2018), with Bonferroni correction for multiple comparisons. Significance was assumed at *P* ≤ 0.05, except for Bonferroni-corrected comparisons, for which the critical value was adjusted accordingly.

## 3 Results and discussion

### 3.1 Improvement in chip design

Fig. 1A shows the 8-channel chip used in these experiments. Fig. 1B shows the recording module design, which improved on a previous design (Lockery et al., 2012) by adding low-profile “side channels” parallel to the “worm channel” that restrains the worm. The side channels permitted a consistent flow of perfusate regardless of how tightly a worm was lodged in the worm channel or worm trap. Perfusate flowed to waste reservoirs (Fig. 1A) via the side-arm channels and worm trap.

### 3.2 Stimulation of pumping by 5HT

The experimental protocol shown in Fig. 2A was used for all experiments unless stated otherwise. After Day-1 adult worms were loaded into a chip, the chip was perfused with a control solution for 30 min. The perfusate was then changed (“switched”) to either a test solution or control solution for an additional 60 min. The eight channels of EPG recordings were acquired continuously for the 90-min period. Time *t* = 0 min was defined as the time at which a solution contacted the worms after the perfusate was switched.

In previous studies (Lockery et al., 2012; Weeks et al., 2018) we perfused *C. elegans* with M9 buffer containing 10 mM 5HT (Raizen and Avery, 1994) to induce robust, sustained pumping. This treatment provides a baseline condition of nearly continuous pumping at ∼4–5 Hz, against which inhibitory effects of anthelmintic drugs can be assessed. To further explore 5HT-stimulated pumping, we investigated its time course and concentration dependence in *C. elegans* N2 worms. Fig. 2B shows representative EPG recordings from seven worms in one chip, before and after switching from M9 to M9 containing 10 mM 5HT. During the baseline period, pumps were infrequent, with small amplitudes. Switching to 10 mM 5HT caused a rapid increase in pump frequency and a more gradual increase in the amplitude of EPG signals. Insets in Fig. 2B illustrate the increase in pump amplitude. Fig. 2C shows pump frequency over time, co-plotted with pump amplitude (the voltage difference between the peaks of the E and R spikes in each pump waveform). Baseline pump amplitude was ∼100 μV, which increased by a factor of 4 by the end of the recording. Baseline pump frequency of <0.5 Hz increased to a steady-state level of ∼4.4 Hz within 15 min of switching to 5HT and was maintained for at least 60 min. In contrast, pump amplitude increased more gradually and may not have reached steady state by 60 min. The *t*_1/2_ (time until the half-maximal value during the observation period was first reached) for pump frequency and amplitude in 10 mM 5HT were 3.6 min and 26.9 min, respectively, an approximately 6-fold difference. We discuss potential causes of the 5HT-stimulated increase in pump amplitude below.

Fig. 2D shows a probability density histogram of IPIs (the time between successive E spikes) in 10 mM 5HT, during steady-state pumping (*t* = 15–60 min). By our definition, an IPI includes the time interval between the E to R spike in a pump (i.e., pump duration) plus the subsequent interval from the R spike to the next E spike. This definition of IPI is synonymous with the “period” of the pumping rhythm. One advantage of this definition compared to others (e.g., Lee et al., 2017) is that IPI or period are the inverse of pump frequency, so values on IPI histograms can be converted directly to pump frequency. IPI histograms indicate the *regularity* of pump timing; a narrow IPI distribution denotes highly regular pumping while a broader distribution denotes an irregular rhythm, with variable gaps between pumps. In a previous study, we found IPI histograms useful in distinguishing EPG phenotypes of *C. elegans* N2s and an Alzheimer's disease model strain (Weeks et al., 2016a). In 10 mM 5HT, the IPI histogram showed a narrow mode (peak) at 225 ms, corresponding to 4.44 Hz (Fig. 2D). The distribution exhibited a shoulder at longer IPIs, but nearly all intervals were <3 s. Thus, the pumping rhythm in 10 mM 5HT was highly stereotyped at ∼4.4 Hz, with occasional short gaps between pumping bouts.

Fig. 2E shows the relationship between 5HT concentration and steady-state pump frequency. The 5HT concentrations used by others to stimulate pumping in intact *C. elegans* generally do not exceed 20 mM (e.g., Hobson et al., 2006; Song and Avery, 2012; Rodríguez-Palero et al., 2018), which was the maximum concentration that we tested. Mean pump frequency increased with 5HT concentration, apparently saturating by ∼10 mM (the concentration used in anthelmintic experiments; see below). The EC_50_ (5HT concentration at which pump frequency reached half-maximal) was calculated to be 1.15 mM. In IPI histograms from 5HT concentrations <10 mM 5HT, the mode remained stable at ∼4.5 Hz while the right shoulder increased as concentration decreased (data not shown). Thus, as 5HT concentration falls, the fundamental pumping rhythm of ∼4.5 Hz continues while gaps become more frequent and of longer duration, to produce a lower mean pump frequency.

For experiments in which we stimulated pumping by perfusing chips with 5HT (above) or OP50 (see Section 3.3), it was important to determine whether worm orientation in worm traps affected responses; specifically, worms lodged head-first might experience reduced sensory stimulation by, and ingest less of, the perfusate. *C. elegans* cuticle is relatively impermeable to solutes and the extent to which particular drugs enter the body across the cuticle vs. by ingestion is generally unknown (e.g., Burns et al., 2010; Ruiz-Lancheros et al., 2011; Law et al., 2015). Law et al. (2015) reported that the EC_50_ of 5HT in a paralysis assay was approximately halved in *bus-17* mutants with enhanced cuticular permeability, indicating that the cuticle normally provides some barrier to 5HT entry. Pharyngeal neurons are located beneath the basal lamina of the pharynx (Avery and You, 2012), so route of entry might influence drugs' influence on these neurons. Two studies of the relative contribution of transcuticular vs. oral entry of IVM in *C. elegans* concluded that ingestion is not required for its anthelmintic effect (Smith and Campbell, 1996; O'Lone and Campbell, 2001). Conversely, the anthelmintic activity of aqueous extracts of bitter melon leaves, *Momordica charantia*, against hookworm host-stage larvae appears to require ingestion (J.C. Weeks, M. Keaney, A. MacIntyre, K.J. Robinson, W.M. Roberts, J.M. Hawdon, unpublished data).

We previously reported that worm orientation had no apparent effect on drug responses (Lockery et al., 2012) but did not test this assumption quantitatively. Data from the experiments shown in Fig. 2 provided an opportunity to do so. Table 1A shows the steady-state pump frequency of worms oriented head-first vs. tail-first in worm traps, for a high (10 mM) and a low (0.1 mM) concentration of 5HT. No significant effect of orientation was detected, even at the low 5HT concentration when this effect might be most pronounced. In contrast, worm orientation did affect pump frequency when OP50 was used as the feeding stimulus (see Section 3.3). Because 5HT is a small molecule, it may have diffused past even tightly-lodged, head-first worms to reach the mouth. Thus, these experiments do not distinguish the relative contribution of transcuticular entry vs. oral ingestion of 5HT in stimulating pumping, which would require better physical isolation of the mouth. Also, the finding that mean pump frequencies did not differ between head-first and tail-first worms indicates that constriction of the head in a worm trap does not inhibit pumping.

**Table 1:** Effect of worm orientation on pump frequency in 5HT and OP50.

		Steady-state pump frequency (Hz; mean ± S.E.M.)		*P* value
Treatment	*n*, worm pairs (*n*, chips)	Head-first worms	Tail-first worms	
**A. [5HT], mM**				
0.1	15 (6)	0.29 ± 0.11	0.73 ± 0.37	0.77
10	18 (8)	3.97 ± 0.16	4.01 ± 0.17	0.64
**B. [OP50], O.D.**_600_				
1	16 (6)	***0.92 ± 0.19***	***1.75 ± 0.35***	0.017^a^
3 to 5	20 (8)	2.61 ± 0.37	2.56 ± 0.32	0.61

Bold italicized font indicates values that differed significantly.

Pairs of head-first and tail-first worms were selected at random from chips in different treatment groups (rows). Beginning ∼30 min after the onset of exposure to (**A**) 5HT or (**B**) OP50 at the concentrations shown, each worm's mean pump frequency was determined over the next 30 min. Pump frequency was compared between pairs of head-first and tail-first worms in the same chip.

a , significant difference in pump frequency between head-first and tail-first worms (1-tailed Wilcoxon signed rank test).

Mechanisms by which 5HT acts rapidly on pharyngeal neural circuitry to stimulate pumping are well described (Song and Avery, 2012; Song et al., 2013; Dallière et al., 2017) and align well with the observed relationship between 5HT concentration and pump frequency in Fig. 2E. However, explaining the progressive increase in EPG voltage amplitude in 10 mM 5HT (Fig. 2B and C) is more complicated. The marked difference in *t*_1/2_ values for pump frequency and amplitude suggested that different mechanisms produced them. During a pump, the amplitude of the electrical current emitted by a muscle (recorded in EPG chips as voltage) is related to the muscle's force of contraction (Robertson et al., 2002; Abongwa et al., 2016). This relationship is seen readily in simultaneous EPG and video recordings, in which the vigor of a pump, and the amplitude of the corresponding EPG waveform, co-vary (data not shown). 5HT receptors are present in pharyngeal muscle as well as neurons (Tsalik et al., 2003; Dempsey et al., 2005; Hobson et al., 2006; Chase and Koelle, 2007) and 5HT shortens action potential duration in pharyngeal muscles, via upstream neurons (Niacaris and Avery, 2003). Franks et al. (2002) reported that when muscle action potentials are eliminated by low-Na^+^ saline, 5HT application reinstates them. However, to our knowledge, none of these mechanisms can account for the increase in pump amplitude over time and its temporal dissociation from increased pump frequency.

Instead, we believe that the slow increase in pump amplitude is caused by changes in *R*_*seal*_, the electrical resistance between a worm's body and the walls of the recording module. Lockery et al. (2012) showed that pump amplitude increases as a worm becomes more deeply seated in the worm trap, which is expected to increase *R*_*seal*_. Two actions of 5HT in *C. elegans* could contribute to an increase in *R*_*seal*_: inhibition of locomotion and stimulation of egg laying. Ranganathan et al. (2000) showed that 33 mM 5HT inhibited locomotion in 100% of *C. elegans* by 10 min and Law et al. (2015) reported that 10 mM 5HT inhibited locomotion (less than 1 body bend/20 s) in ∼40% of worms by 30 min. If a worm's normal “swimming” movements in liquid medium tend to push it away from the narrow funnel at the head of the worm trap (Fig. 1B), then immobilization could allow perfusate flowing past the worm to push it deeper into the trap and thereby increase *R*_*seal*_. Waggoner et al. (2000) and Carnell et al. (2005) reported that 10–12.5 mM 5HT caused a >10-fold increase in egg laying within 60–90 min; accumulation of eggs in the spaces between a worm's body and the channel walls, making the fit tighter, could likewise increase *R*_*seal*_. We did not measure *R*_*seal*_ in 5HT experiments, but measurements provided in Section 3.6 support the hypothesis that an increase in *R*_*seal*_ causes increased EPG amplitude.

Variations in R_seal_ during experiments was one of the motivations for abandoning a previous analysis method that quantified the magnitude of rectified EPG signals as the readout for drug effects (Lockery et al., 2012). In contrast, our improved software algorithm extracts EPG frequency independently of changes in signal amplitude, until the signal-to-noise ratio becomes too small to distinguish spikes from noise. With modifications to the amplifier hardware and software, it is also now possible to record EPG currents rather than voltages and eliminate any effects of changes in *R*_*seal*_ (W.M. Roberts, J.C. Weeks and S.R. Lockery, personal communication).

### 3.3 Stimulation of pumping by *E. coli* OP50

Although 5HT is a convenient stimulator of robust, sustained pharyngeal pumping, its use is sometimes undesirable: e.g., when investigating the activation of feeding behavior by natural stimuli. Katzen (2017) and Lee et al. (2017) showed that bacterial suspensions perfused through microfluidic devices can stimulate pumping, and we investigated this phenomenon in the 8-channel EPG chip using *C. elegans*’ usual laboratory food source, *E. coli* strain OP50. OP50 was suspended in M9 at a range of concentrations measured by optical density at 600 nm (O.D._600_) (see Section 2.4). Before starting this experimental series, we confirmed that bacterial suspensions did not clog microfluidic channels by including Fast Green in the perfusate to visualize flow (Lockery et al., 2012). A syringe filled with OP50 (O.D._600_ = 5, in M9 with 0.005% Fast Green) sat horizontally on the syringe pump for 30 min (our usual protocol; Fig. 2A) before being used to perfuse worms for 90 min. We detected no channel blockage or leakage (*n* = 3 chips; data not shown). We also confirmed that bacteria did not settle out of suspension during experiments, which could reduce the concentration that worms experience. This was done by measuring the O.D._600_ of the perfusate before loading it on the syringe pump and after it had passed through the recording channels and accumulated in waste reservoirs. The mean O.D._600_ of perfusate from the reservoirs was 98% of its initial value and the values did not differ significantly [before perfusion, 4.2 ± 0.3 (O.D._600_ mean ± S.E.M.); after perfusion, 4.2 ± 0.4; *n* = 6 chips, Wilcoxon Signed-Rank Test, *P* > 0.05]. We conclude that bacteria remained in suspension during experiments, at their pre-perfusion concentration, for at least 120 min.

Fig. 3A shows EPGs from seven worms in one chip before and after switching perfusate from M9 alone to OP50 (O.D._600_ = 4) in M9. In all experiments, worms were food-deprived for 2 h prior to the switch to OP50, which reduces pumping to low levels and enhances the response when food is again provided (Lemieux and Ashrafi, 2015; Dallière et al., 2016). During the baseline period, pumping occurred at low frequency. The switch to OP50 caused pumping to increase but—unlike the nearly continuous pumping observed in 10 mM 5HT—the pumping occurred in bouts, with long pump-free intervals between. This aspect is quantified below using IPI analysis.

Fig. 3B shows the concentration-dependence and time course of OP50-stimulated pumping. Mean pump frequency during the baseline period was <0.5 Hz in all groups. OP50 induced a biphasic response, in which pump frequency was highest during the first ∼15 min (“rapid” response) and then declined to a lower, sustained level (“sustained” response). The rapid and sustained responses to OP50 are displayed as Hill plots in Fig. 3C. The highest four OP50 concentrations (O.D._600_ = 2, 3, 4 or 5) appeared saturating in their ability to stimulate pumping. For these four groups, there were no significant differences between mean pump frequencies during the rapid responses (*t* = 2–7 min), nor during the sustained responses (*t* = 30–60 min) [one-way ANOVA, (*F*(3,85) = 1.14, *P* = 0.38) and (*F*(3,85) = 1.06, *P* = 0.98), respectively; *n* = 19–33 worms/group]. Similarly, Lee et al. (2017) reported saturation of pump frequency at O.D._600_ = 3 and above, measured in a microfluidic device. Accordingly, values from these four groups (O.D._600_ = 2–5) were combined; the mean pump frequency during the rapid and sustained responses to OP50 differed significantly [repeated-measures ANOVA, (*F*(1,88) = 24.4, *P* < 0.0001); *n* = 141 worms]. The underlying mechanism(s) of the biphasic behavioral response to OP50 is unknown. One possibility is sensory adaptation in olfactory neurons that detect the sudden presence of food: e.g., the *C. elegans* neuron AWA, which is sensitive to food-related odors and responds to odor in an initial transient that decays to a plateau (Larsch et al., 2015). Another possibility is that pump frequency falls because worms become partially satiated.

A positive relationship between food concentration and pump frequency in *C. elegans* (Fig. 3B and C) is well known (e.g., Avery and Horvitz, 1990; Lee et al., 2017). However, temporal features revealed by continuous EPG recordings may have been missed previously. Most investigators measure pump frequency by counting pumps by eye and dividing by the observation time (typically, 60 s or less). For time-series data, counts are made at intervals rather than continuously. For example, counting pumps for 10 s by eye, Lemieux et al. (2015) reported that worms re-fed after 2 h of food deprivation had significantly increased pump frequency 5, 30 and 60 min after re-feeding, with similar frequencies at the three time points. We likewise saw that pump frequency remained elevated for at least 60 min after re-feeding (Fig. 3B, D) but observed a significant elevation at 5 min that was not reported by Lemieux et al. (2015). More experiments would be required to reconcile this difference. One potential factor is that worms perfused with OP50 in microfluidic channels experience a different sensory environment than worms placed on bacterial lawns. Another, and quite likely, possibility is that counting pumps by eye for short periods misses finer temporal details of pharyngeal pumping. EPGs provide a continuous, millisecond-resolution record of pumping behavior but in a less naturalistic environment than an agar plate. Depending on an investigator's goals, one or the other method may be more appropriate. Limitations of visual counting of pumping during short observation periods are further discussed in Scholz et al. (2016).

The maximum pump frequency in OP50 was lower than in 5HT. Mean pump frequencies for saturating concentrations of OP50 (O.D._600_ = 2–5) were 3.28 ± 0.14 Hz for the rapid, and 2.54 ± 0.13 Hz for the sustained, responses (S.E.M., *n* = 141 worms), whereas maximum pump frequencies in 5HT were 4.52 ± 0.08 Hz, and 4.59 ± 0.09 Hz, for 10 and 20 mM 5HT, respectively (*n* = 23 worms/group; Fig. 2E). The maximum post-switch recording period analyzed in this study was 60 min, but steady-state pumping in 10 mM 5HT continues for at least 90 min (data not shown). Lockery et al. (2012) reported that N2s continue pumping in 10 mM 5HT for at least 6–8 h, but did not quantify pump frequency over extended periods. We did not examine pump frequency in worms perfused with OP50 for longer than 60 min.

Importantly, in contrast to the marked increase in pump amplitude in 10 mM 5HT over time (Fig. 2C), pump amplitude remained stable at ∼100 μV in OP50 (Fig. 3D). Sensory cues from bacteria and greater food abundance increase egg laying, but less dramatically than for 5HT treatment (Waggoner et al., 2000; Schafer, 2005; Harvey and Orbidans, 2011), and food-deprived worms transferred to bacteria on agar plates show a locomotory slowing response (Sawin et al., 2000). Video-recording worms during perfusion with 5HT or OP50 could reveal differences in egg laying and locomotion that influence their position in worm traps and hence *R*_*seal*_. Regardless, the data in Fig. 2, Fig. 3D suggest that pump amplitudes measured in OP50 are less subject to error from changes in *R*_*seal*_ than are amplitudes measured in 5HT. As discussed above, measuring EPG currents rather than voltages would eliminate changes in *R*_*seal*_ as a confounding factor in future experiments.

Fig. 3E shows an IPI probability density histogram for saturating concentrations of OP50, from *t* = 30–60 min. The mode was at 205 ms, corresponding to 4.88 Hz, but there was also an extensive shoulder at longer IPIs, with the probability density remaining elevated at even 10 s (and beyond; data not shown). This result contrasts markedly with the IPI histogram for 10 mM 5HT (Fig. 2D), in which the shoulder of the histogram reached an unmeasurably low level by 3–4 s. However, a lower 5HT concentration (1 mM) that produced a mean steady-state pump frequency similar that produced by the saturating concentrations of OP50 (2–3 Hz; Fig. 2, Fig. 3B) had an IPI distribution nearly identical to that in Fig. 3E (data not shown). Therefore, the observed differences between the pattern of pumping stimulated by high concentrations of 5HT and OP50 may be explained by the differences in the maximal level of pumping that these different stimuli evoke. In OP50, pump frequency during bouts was similar to that seen in 10 mM 5HT (i.e., 4–5 Hz), as shown by the similar modes of the IPI histograms for 10 mM 5HT and saturating concentrations of OP50 (Fig. 2, Fig. 3E). However, the more frequent occurrence of pump-free gaps in OP50, and their increased lengths, drove down the mean pump frequency compared to 10 mM 5HT. As discussed in Lee et al. (2017), *C. elegans* pump steadily on dense bacterial lawns but, at lower or patchy food concentrations (which may better resemble their natural environment), worms pump in bouts separated by gaps. We suggest that the temporal patterning of pumping in EPG chips stimulated by OP50, or moderate concentrations of 5HT (e.g., 1 mM), may better resemble *C. elegans* behavior in natural environments than the clock-like pumping stimulated by 10 mM 5HT.

Sensory receptors that stimulate feeding are located in the head region (Dallière et al., 2017), so we tested whether head-first vs. tail-first orientation of worms in worm traps affected their responses to OP50. Table 1B compares sustained pump frequency (*t* = 30–60 min after perfusate switch) in worms exposed to low or high concentrations of OP50 (O.D._600_ = 1, or 3–5, respectively). For the high concentration, worm orientation had no significant effect on pump frequency. In contrast, in the low OP50 concentration, tail-first worms (whose heads were directed into the perfusate flow) responded with significantly higher pump frequency than did head-first worms (Table 1B); this result suggests that worms positioned head-first into worm traps experience reduced feeding-stimulating sensory stimuli, but that this effect is significant only in low bacterial concentrations. In contrast, worm orientation did not affect pump frequency in 5HT, regardless of concentration (Table 1A).

When loading EPG chips, worms orient more or less randomly into head-first or tail-first orientation in worm traps (data not shown). In the 8-channel chip (Fig. 1), worms can be manipulated in the distribution network while loading until they assume the desired orientation, but the additional handling can damage them, especially in strains that are fragile or sticky (see Section 3.5). Alternatively, worms could be loaded randomly and data from those in the “wrong” orientation discarded. For experiments in which high reproducibility is desired when using dilute bacterial suspensions, one could incorporate chip design features that position worms in one orientation (Hu et al., 2013; Ardeshiri et al., 2016). The issue of worm orientation can be avoided by using higher OP50 concentrations (O.D._600_ ≥ 3).

In experiments using EPG recordings as a readout of anthelmintic activity (Sections 3.4 to 3.7), we used 10 mM 5HT as the pumping stimulus, to provide a high level of baseline activity against which inhibitory effects could be detected. We have not yet tested anthelmintics on worms stimulated to pump by OP50. Potentially, the lower baseline activity and more irregular pumping evoked by OP50 could reduce the sensitivity of EPG recordings in detecting anthelmintic activity. However, host-stage larvae of *A. ceylanicum* and *A. suum* stimulated to pump in EPG chips by blood serum or 5HT have baseline pump frequencies of ∼1 Hz and pump inhibition by IVM is readily detected at concentrations spanning several orders of magnitude (Weeks et al., 2016b).

In summary, perfusion of 5HT or OP50 through 8-channel EPG chips stimulates sustained (>60 min) pumping in *C. elegans*, but with different temporal features and concentration dependence (Fig. 2, Fig. 3). The above comparison of the two methods provides a framework for investigators to select the most appropriate pumping stimulus based on experimental objectives.

### 3.4 Ivermectin (IVM) effects on susceptible and resistant *C. elegans*

For subsequent experiments, all EPG recordings were performed in 10 mM 5HT in M9 (henceforth termed “M9-5HT”), to induce robust, sustained pumping suitable for detecting inhibitory effects of applied drugs (Lockery et al., 2012; Weeks et al., 2018). Worms were pre-incubated in M9-5HT for ≥10 min before loading, to accommodate the latency to reach steady-state pump frequency (Fig. 2C). After a 30 min baseline recording period, the perfusate was switched to a test solution (Fig. 2A). The perfusate switch occurred at least 45 min after the onset of 5HT exposure, at which time pump amplitude would still be increasing (Fig. 2C). Accordingly, pump amplitude data is not presented below for anthelmintic experiments, with one exception (see Section 3.6).

We first tested the effects of the anthelmintic drug, ivermectin (IVM). The IVM receptor, GluCl, mediates synaptic inhibition from motoneuron M3 onto pharyngeal muscles; these synapses are active between the E and R spikes to help terminate each pump (Avery and You, 2012). As a GluCl agonist, IVM causes flaccid paralysis of the body and pharynx, but may have additional anthelmintic effects, particularly in parasitic nematodes (Wolstenholme and Rogers, 2005; Hernando and Bouzat, 2014; Wolstenholme et al., 2016b). The inhibition of pharyngeal pumping by IVM has been reported previously in *C. elegans* and other nematodes (Avery and Horvitz, 1990; Geary et al., 1993; Holden-Dye and Walker, 2006). We compared N2 worms with a triple IVM receptor subunit knock-out strain, *avr-14*; *avr-15*; *glc-1*, which is highly resistant to IVM (Dent et al., 2000). Lockery et al. (2012) published microfluidic EPG recordings from these strains in IVM but with small sample sizes, a single IVM concentration and without the improved spike recognition and data analysis software used here.

Fig. 4A shows representative EPG recordings of individual N2 worms while switching perfusate from M9-5HT to either M9-5HT (control experiments) or to M9-5HT with 3 μM IVM. In control experiments (Fig. 4Ai, Aii), the perfusate switch had no obvious effect on EPG activity in worms of either strain. When the N2 worm was switched to 3 μM IVM (Fig. 4Aiii), pumping ceased within ∼15 min. In contrast, in 3 μM IVM, the *avr-14*; *avr-15*; *glc-1* worm continued pumping long after the N2 worm had stopped (Fig. 4Aiv). Pump frequency in the *avr-14*; *avr-15*; *glc-1* worm initially appeared unaffected by the switch but then decreased, with pump-free gaps appearing. These observations confirm that *avr-14*; *avr-15*; *glc-1* worms are less susceptible than N2s to the anthelmintic activity of IVM, as assayed by EPG recordings. For the reason given above, we did not analyze pump amplitudes quantitatively, but the decrease in pump amplitude in N2s immediately prior to pumping cessation in IVM (Fig. 4Aiii) was reported previously (Lockery et al., 2012).

Fig. 4B displays pump frequency over time in groups of N2 worms switched to different concentrations of IVM, while Fig. 4C shows parallel data for *avr-14*; *avr-15*; *glc-1* worms. One immediate difference was in baseline pump frequency, which was significantly lower in *avr-14*; *avr-15*; *glc-1* worms vs. N2s (*avr-14*; *avr-15*; *glc-1*, 3.29 ± 0.04 Hz, *n* = 96 worms; N2, 4.02 ± 0.07 Hz, *n* = 136 worms; *P* < 0.0001, 2-tailed Mann-Whitney *U* test; the same test was used for all subsequent comparisons of baseline pump frequency). The cause of this difference is unknown, but mutations in many genes can alter worms’ pump basal frequencies; a search of the CGC website using the term “pumping” in the “description” field yields more than 40 genotypes with slow, fast or irregular pumping phenotypes. To compare strains with dissimilar baseline pump frequencies, we normalized pump frequency to its mean value during a 10 min interval (*t* = −12 to −2 min) during the baseline period (see Section 2.6; Weeks et al., 2016b; Weeks et al., 2018). In Fig. 4B and C, and in the corresponding normalized data (Fig. 4D), N2 and *avr-14*; *avr-15*; *glc-1* worms switched to M9-5HT (control experiments) maintained a steady frequency for the 60 min recording period whereas switching to IVM caused concentration-dependent inhibition of pumping in both strains. The superimposed, normalized data in Fig. 4D show that the pump frequency plots of *avr-14*; *avr-15*; *glc-1* worms were right-shifted (indicating drug resistance) compared to N2s for the four IVM concentrations tested.

For statistical comparisons of these data, we computed the cumulative fraction (CF) of pumps that occurred between drug onset (*t* = 0 min) and the end of the experiment (*t* = 60 min), with *CF*_50_ being the time at which 50% of the pumps had occurred (Weeks et al., 2016b). In Fig. 4E, CF plots from the N2 and *avr-14*; *avr-15*; *glc-1* controls (black traces, no IVM) rose approximately linearly, indicating a steady rate of pumping. Unexpectedly, unlike other *CF*_50_ comparisons in this study (Fig. 6, Fig. 9E), the *CF*_50_ values for N2 and mutant controls differed significantly (*P* < 0.01; 2-tailed Wilcoxon Mann-Whitney *U* test, used for all *CF*_50_ statistical comparisons). As seen in Fig. 4E, the CF plot of *avr-14*; *avr-15*; *glc-1* controls was slightly right-shifted compared to N2s, due to a small increase in pump frequency in *avr-14*; *avr-15*; *glc-1* worms relative to N2 worms during the final ∼15 min of the EPG recording (Fig. 4D). We did not correct for this difference because it occurred near the end of the recording period and would have reduced, not enhanced, the measured resistance of *avr-14*; *avr-15*; *glc-1* vs. N2 worms. When perfused with IVM, the *CF*_50_ values of N2 and *avr-14*; *avr-15*; *glc-1* worms differed significantly at all concentrations tested (0.1 μM, *P* < 0.005; 1 μM, *P* < 0.001; 3 μM, *P* < 10^−6^; 10 μM, *P* < 10^−6^; Fig. 4E).

These data confirm the expected IVM-resistant phenotype of *avr-14; avr-15; glc-1* worms, assayed by EPG recordings. It should be noted that, depending on the shape of CF plots, it may be advantageous to compare CF values at a higher intercept (e.g., *CF*_75_), in addition to or instead of *CF*_50_; e.g., anthelmintic drug efficacy studies often compare multiple EC (effective concentration) values (e.g., EC_50_, EC_95_; Keiser et al., 2009). We compared *CF*_50_ values in the present study to match previous work (Weeks et al., 2016b).

Distinguishing drug-sensitive and drug-resistant worms is important for basic research (e.g., forward genetic screens; Somvanshi et al., 2014) as well as veterinary and medical testing of natural worm populations for drug resistance (Wolstenholme et al., 2004). In addition to comparing isogenic *C. elegans* strains with different drug sensitivities (e.g., Fig. 4), it is feasible to record EPGs from mixed populations of worms and then recover individuals of interest. The 8-channel chip was not designed specifically for this capability but it is not difficult to penetrate the PDMS layer of a chip with a syringe needle and recover a worm from a channel (https://nemametrix.com/product/worm-recovery-kit/).

The EC_50_ for GluCl activation in dissected pharynges of *C. elegans* is 2–3 nM IVM (Pemberton et al., 2001; Holden-Dye and Walker, 2006) but the drug permeability barrier of the *C. elegans* cuticle (see Section 3.5) requires higher concentrations and/or longer exposures compared to exposed tissue. The IVM concentrations effective in our 60-min treatment protocol were 0.1–10 μM, similar to or greater than those typically reported for intact *C. elegans*. For example, the EC_50_ for motility assays is reported as ∼1 μM IVM for treatment durations of 1 or 18 h (Glendinning et al., 2011; Janssen et al., 2015) whereas Ardelli et al. (2009) reported that nanomolar concentrations of IVM inhibited pharyngeal pumping and motility after 2.5 h. Three factors are relevant to the drug concentrations used in our experiments. First, to increase throughput, we used concentrations that inhibit pumping within a 60-min post-switch interval. Second, PDMS absorbs small molecules, thereby reducing their concentration in solution (Moraes et al., 2012). Using HPLC, we determined that ∼80% of the IVM in a 10 μM solution is lost after a 60-min perfusion through the 8-channel chip (A. Moghaddam, K.J. Robinson and J.C. Weeks, unpublished data). Thus, worms likely experienced IVM concentrations 5-fold lower than reported here. We did not perform HPLC on other drugs used in this study (e.g., 5HT) but their concentrations were likely also reduced. This issue can be reduced or eliminated by pretreating PDMS devices with molecules that block absorption or fabricating chips from glass or hard plastic (e.g., Gomez-Sjoberg et al., 2010; Ren et al., 2011). Finally, we have not explored whether using 10 mM 5HT to drive maximal pumping frequencies in baseline conditions might reduce sensitivity to applied anthelmintic drugs, perhaps requiring the use of higher concentrations to counteract the strong pumping stimulus.

Our 60-min post-switch recording protocol has been successful for drugs with rapid actions such as IVM, LEV (Section 3.6) and PPZ (Section 3.7) but longer observation periods may be required for drugs with slower modes of action. Worms perfused with 10 mM 5HT continue to pump for at least 6–8 h (Lockery et al., 2012) so, with appropriate controls, it should be possible to detect anthelmintic effects on pumping over substantially longer recording periods than the ones reported here. Conversely, it can be desirable to shorten the time required for each experiment. Based on our experience, reducing the baseline EPG recording period from 30 to 15 min should have no appreciable effect on experimental results (data not shown).

EPG recordings reveal additional features of anthelmintic effects on pharyngeal pumping beyond pump frequency. Fig. 5Ai shows an IPI histogram for N2 worms during the 60-min post-switch interval, for different concentrations of IVM. The mode of the distribution was maintained with high fidelity in all groups (range, 205–225 ms; or 4.9 to 4.4 Hz), whereas the right shoulder of the distribution increased with increasing IVM concentration, indicating an increased probability of long gaps. Thus, the decrease in mean pump frequency evoked by IVM (Fig. 4) results from increasing interruption of the normal pumping rhythm by gaps, until pumping ceases (Lockery et al., 2012). Fig. 5B shows data on pump duration. In control N2 worms, pump duration was quite stable during the baseline and post-switch period. For an intermediate IVM concentration (1 μM) that did not terminate pumping, pump duration was largely maintained, but with more variability than in controls. In higher IVM concentrations that terminate pumping, pump duration decreases dramatically prior to cessation (Lockery et al., 2012; data not shown).

### 3.5 Effects of cuticle permeability on ivermectin sensitivity

Various approaches have been taken to overcome the permeability barrier of the *C. elegans* cuticle: e.g., the use of mutants with defects in the epidermis and cuticle, and incubating worms in hypotonic solutions to disrupt the barrier (Gürel et al., 2012; Law et al., 2015; Kudelska et al., 2017). As proof of principle that permeability mutants can be used in microfluidic chips, we compared the IVM sensitivity of N2s to a representative permeability mutant, *bus-8* (glycosyltransferase; Partridge et al., 2008). Fig. 6A shows representative EPG recordings of individual worms. In control experiments with both strains (Fig. 6Ai, ii), EPG activity continued throughout the recording period. Switching to 3 μM IVM (Fig. 6Aiii, iv) caused pump amplitude to decrease and pumping to cease, as also seen in Fig. 4Aiii and Lockery et al. (2012). However, pumping ceased more rapidly in *bus-8* than in N2 worms, consistent with enhanced IVM access.

Normalized group data appear in Fig. 6B. Baseline pump frequency did not differ significantly between *bus-8* and N2 worms (*bus-8*, 4.18 ± 0.12 Hz, *n* = 46 worms; N2, 4.47 ± 0.08 Hz, *n* = 50 worms; *P* = 0.11). In these experiments, *bus-8* worms could potentially have shown increased baseline pump frequency due to enhanced 5HT entry across the cuticle (although steady-state pump frequency saturates by 10 mM 5HT; Fig. 2E) but we did not observe this. Pump frequency in N2s during the baseline and post-switch periods was similar to that in other experiments (Fig. 4, Fig. 7, Fig. 9B), suggesting that pre-treatment of chips with Pluronic F-127 (to reduce adhesion between *bus-8* worms and PDMS; see Section 2.5) was not toxic to N2 worms. In contrast, the pump frequency of *bus-8* worms in control conditions declined beginning ∼30 min post-switch (Fig. 6B); possibly, they suffered damage during loading or Pluronic F-127 had a delayed, deleterious effect.

After switching to 3 μM IVM, pump frequency decreased in both strains, but more rapidly in *bus-8* than in N2s; e.g., the time to reach 50% of baseline pump frequency was 37% shorter in *bus-8* than N2s (*bus-8*, 8.8 min; N2, 13.9 min; Fig. 6B). Fig. 6C shows CF data for the two strains. *CF*_*50*_ values in control conditions did not differ significantly (*P* = 0.07) whereas the *CF*_*50*_ value in 3 μM IVM was significantly reduced in *bus-8* compared to N2 worms (*P* < 0.002). These results are consistent with the *bus-8* mutation increasing worms’ sensitivity to 3 μM IVM, as assessed by inhibition of pharyngeal pumping. A similar result was obtained when 1 μM IVM was tested (data not shown).

A number of studies have assessed the utility of *C. elegans* mutants with enhanced permeability for drug and toxicology testing. When comparing N2s with a *bus-17* allele in a motility assay, Kudelska et al. (2017) found that EC_50_ values for two nicotinic compounds were reduced 5- to 8-fold in the permeability mutant; the *bus-17* mutant also revealed paralytic effects of two other compounds that were ineffective in N2s. Gürel et al. (2012) reported that dissolving 5HT in water rather than M9 increased its ability to paralyze worms by ∼30-fold. When screening the NIH Clinical Collection library for anthelmintic activity, Weeks et al. (2018) found *bus-8* to be no more sensitive than N2s in lethality and growth inhibition assays. Xiong et al. (2017) assessed *C. elegans* permeability mutants for use in toxicology testing. All mutants showed decreased fitness (based on development rate and brood size), with a *bus-5* mutant providing the best combination of sensitivity and fitness in their protocol. In the current experiments, the modest increase in sensitivity to IVM in *bus-8* worms (Fig. 6) was somewhat offset by the need to pretreat microfluidic chips with Pluronic F-127 and the unexplained decreased in pump frequency in the *bus-8* control worms (Fig. 6B). In the future, it would be prudent to adopt the strategy of Xiong et al. (2017) and test a panel of cuticle permeability mutants to identify the strain(s) best suited for experiments using PDMS microfluidic chips.

### 3.6 Levamisole effects on susceptible and resistant *C. elegans*

We next tested the effects of LEV, an agonist of levamisole-sensitive acetylcholine receptors (L-AChRs) in *C. elegans* and other nematodes (Towers et al., 2005; Martin and Robertson, 2010; Holden-Dye and Walker, 2014; Blanchard et al., 2018). Activation of these receptors causes spastic (hyper-contracted) paralysis. A deletion mutant of the L-AChR subunit UNC-38, *unc-38,* is less sensitive to LEV than N2s in paralysis assays (Sloan et al., 2015). Significantly for the present study, L-AChRs are present on *C. elegans* body wall muscles but absent from the pharynx, are not required for pharyngeal pumping, and LEV does not affect pharyngeal pumping in exposed *C. elegans* pharynges (Avery, 1993; Fleming et al., 1997; Culetto et al., 2004; Towers et al., 2005). The absence of L-AChRs from the pharynx allowed us to investigate the ability of EPG recordings to detect extra-pharyngeal anthelmintic actions in intact worms. Lockery et al. (2012) reported that 10 mM LEV inhibited EPG activity in N2s and the IVM-resistant mutant *avr-14*; *avr-15*; *glc-1*, but did not test a LEV-resistant mutant nor explore the concentration dependence of LEV actions.

Fig. 7A shows EPG recordings of N2 and *unc-38* worms. In control experiments (Fig. 7Ai,ii), EPG activity continued steadily for the entire recording period; pumping appeared somewhat more irregular in *unc-38* worms. When switched to 3 mM LEV, the N2 worm (Fig. 7Aiii) showed a rapid, transient inhibition of pumping followed by partial recovery to a pump frequency lower than baseline. Inhibition of pumping in *unc-38* worms (Fig. 7Aiv) by LEV appeared weaker than in N2s. It should be noted that the disruption of pumping by LEV in both N2 and *unc-38* worms was considerably more variable (data not shown) than the rather stereotyped responses to IVM (Fig. 4, Fig. 6; Lockery et al., 2012). The EPG traces in Fig. 7Aiii,iv do not capture the full variety of responses to LEV observed.

Fig. 7B and C displays mean pump frequency over time in groups of N2 and *unc-38* worms switched to different concentrations of LEV. As discussed above for IVM, the LEV concentrations experienced by worms in these experiments may have been reduced by absorption into PDMS. Baseline pump frequency was slightly reduced in *unc-38* worms compared to N2s (*unc-38*, 3.65 ± 0.06 Hz, *n* = 113 worms; N2, 3.88 ± 0.06 Hz, *n* = 113 worms; *P* < 0.01). Towers et al. (2005) likewise reported a reduced basal pump frequency in *lev-8,* a LEV-resistant mutant with a defective L-AChR subunit. Control N2 and *unc-38* worms maintained steady pumping during the post-switch period (Fig. 7B and C). In N2s, switching to LEV caused a concentration-dependent inhibition of pumping with two phases: first, a rapid inhibition during the first ∼15 min, followed by a partial recovery (Fig. 7B). The two phases were most apparent at intermediate concentrations of LEV (0.3 mM and 1 mM). In contrast, LEV-induced inhibition of pumping in *unc-38* lacked the rapid phase of inhibition and instead showed a smooth decline to a lower pump frequency, which was maintained (Fig. 7C). The latter response was more similar to the inhibition of pumping by IVM (Fig. 4, Fig. 6). Normalized data in Fig. 7D highlight the qualitatively different temporal pattern of inhibition in the two strains and illustrate the decreased sensitivity of *unc-38* to LEV compared to N2s, as expected.

The difference in pump inhibition kinetics in N2 and *unc-38* worms made CF_50_ plots problematic. Instead, we compared pump frequency during two intervals during the post-switch period: *t* = 0–15 min to capture the “rapid” inhibition by LEV and *t* = 45–60 min for the “sustained” inhibition. Fig. 7E shows these data fitted using the Hill equation, from which EC_50_ values were derived (see Figure Caption). The EC_50_ value for rapid inhibition in N2s differed significantly from those of the other three categories of inhibition: N2 sustained, *unc-38* rapid and *unc-38* sustained [all *P* < 2 × 10^−5^; all EC_50_ comparisons by a Likelihood Ratio test (Weeks et al., 2018) with Bonferroni correction for multiple comparisons], while the latter three EC_50_ values did not differ significantly from each other (*P* = 0.32 and 0.028, critical value of *P* adjusted to 0.009 by Bonferroni correction). Thus, Fig. 7 shows that (1) the rapid phase of pump inhibition produced by LEV was unique to N2s and absent in *unc-38* worms, and (2) by *t* = 45–60 min post-switch, the reduction in pump frequency was similar in the susceptible and resistant strains. Thus, LEV resistance was manifested only during an initial phase of pump inhibition.

Fig. 5Aii shows IPI data for N2 worms treated with LEV. The modal value of the IPI probability density histogram was similar in 0 and 0.1 mM LEV (235 and 215 ms; 4.3 and 4.7 Hz), greater in 1 mM LEV (285 ms; 3.5 Hz) and the greatest in 3 and 10 mM LEV (375 and 505 ms; 2.7 and 2.0 Hz, respectively). A second notable feature of the histogram was increasing height of the right shoulder with LEV concentration. Thus, both a decrease in modal pump frequency, and increased probability of long IPIs, contributed to the concentration-dependent decrease in pump frequency produced by LEV (Fig. 7B). This pattern contrasts with both IVM (Fig. 5Ai) and PPZ (see Section 3.7; Fig. 5Aiii).

Fig. 5B shows that, in N2 worms (solid pink line), an intermediate LEV concentration (1 mM) caused an initial increase in pump duration, followed by partial recovery to a sustained but elevated level. The early, rapid inhibition of pumping in LEV was present in N2 but not *unc-38* worms (Fig. 7), and we likewise found that the initial increase in pump duration was reduced in *unc-38* worms (Fig. 5B, dashed pink line). This finding suggests that increased pump duration and decreased pump frequency in LEV may be related, but we did not explore this possibility. Interpreting these results in terms of LEV action on L-AChRs is complicated by the fact that these receptors are absent from the pharynx and the canonical action of LEV is to paralyze body wall muscles. Intriguingly, Takahashi and Takagi (2017) showed that optogenetic silencing of *C. elegans* body wall muscles likewise inhibits pharyngeal pumping and, from genetic manipulations, propose that independent neural and neurohumoral pathways contribute. These authors suggest that body-wall relaxation could activate proprioceptive or mechanoreceptive signals, or stimulate the release of retrograde signals from the muscles, that act via the proposed pathways to inhibit neural circuitry for pumping. Further experiments are needed to better elucidate this phenomenon. Regardless, our data confirm the ability of EPG recordings to detect extra-pharyngeal anthelmintic actions in intact worms.

As with IVM, elevated concentrations of LEV were required to elicit rapid electrophysiological responses and help overcome potential drug absorption into PDMS. Effective concentrations for LEV-induced paralysis are quite variable. Sloan et al. (2015) reported that 0.01 mM LEV caused paralysis in 96% of N2, and 33% of *unc-38*, worms after 45 min. Other studies report a paralysis EC_50_ of 9 μM LEV after a 60 min exposure, and that 1 mM LEV requires 75–90 min for full paralysis (Gottschalk et al., 2005; Qian et al., 2008). In a microfluidic device containing PDMS, Ding et al. (2017) reported an EC_50_ of 2.23 μM LEV for the inhibition of motility.

These experiments revealed an unexpected aspect of LEV action. The dimensions of channel features in the EPG chip are optimized to ensure that Day-1 adult N2s lodge in the worm trap and rarely slip through. However, a relatively high proportion of LEV-treated worms were expelled through the worm trap into waste reservoirs (see Fig. 1B). Table 2 shows that the percentage of expelled N2 worms increased with LEV concentration, from 5.4% of controls to 43.6% of worms in 10 mM LEV (*P* = 0.0001; 2-tailed Fisher's Exact Test), and that the expulsion rate in 10 mM LEV was greater than for all other concentrations (P < 0.03). Importantly, *unc-38* worms were significantly less likely to be expelled than N2s; e.g., only 10.7% of *unc-38* worms in 10 mM LEV were expelled compared to 43.6% of N2s (*P* < 0.01). This phenomenon was not observed with other anthelmintics; e.g., in the highest IVM concentration tested (10 μM IVM) only 1.9% (1 of 52) of N2 worms were expelled. In the highest PPZ concentration tested (100 mM), 18.9% (7 of 37) of N2 worms were expelled compared to 4.7% (1 of 21) of controls, but this difference did not reach significance (*P* = 0.24). Thus, only LEV increased the proportion of worms that slipped through the worm trap; the possible cause of this difference is discussed below.

**Table 2:** Expulsion of worms through the worm trap in N2 and *unc-38* (LEV-resistant) worms.

[LEV], mM	Strain			
	N2		*unc-38* (LEV-resistant)	
	% expelled	*n* (worms)	% expelled	*n* (worms)
0	5.4	56	2.9	34
0.1	13.0	23	4.3	23
0.3	16.7	24	4.3	23
1.0	10.0	30	7.1	28
3.0	13.3	30	7.1	28
10	***43.6***^a^^,^^b^	39	10.7	28

Value that differed significantly from comparator groups shown bold and italicized.

a % expelled in 10 mM LEV differs significantly from worms of the same genotype treated with all other LEV concentrations (Fisher's Exact test, 2-tailed; *P* = 0.0001 to 0.03).

b Value differs significantly from corresponding group of *unc-63* worms treated with the same LEV concentration (*P* = 0.006).

Worm expulsion proved unexpectedly useful in investigating the effect of R_seal_ on EPG amplitude. As worms slipped farther into the trap and became tightly compressed, the amplitude of their EPG signals increased dramatically; e.g., in the example shown in Fig. 8, peak-to-peak EPG amplitude increased 16-fold, followed by abrupt loss of the signal when the worm exited the recording module into the waste reservoir. We hypothesized above (Section 3.2) that R_seal_ increases as a worm is propelled deeper into the trap, which might explain the increase in pump amplitude over time in 5HT. To test this hypothesis, we measured the RMS amplitude of high-frequency noise in EPG recordings during LEV experiments to provide a proxy of R_seal_ (see Section 2.7). RMS noise (mean ± S.E.M.; *n* = 5 worms from 3 chips) was 6.5 ± 0.21 μV during the baseline period (“PRE”), 13.9 ± 5.1 μV during the time of maximum EPG amplitude (“MAX”), and 6.04 ± 0.21 μV after worms were expelled (“POST”). PRE and POST noise values did not differ significantly (*P* = 0.21; 2-tailed Mann-Whitney test) whereas the MAX noise value differed significantly from both PRE and POST values (*P* < 0.01). These data provide strong evidence that worm position in the worm trap alters R_seal_ and, artifactually, EPG amplitude. As discussed above (Section 3.2), 5HT stimulates egg laying, which could also increase R_seal_; LEV likewise stimulates egg laying in *C. elegans* (Towers et al., 2005), while IVM inhibits egg laying (Yan et al., 2012) and we could not find any published data on piperazine. Because the 10 mM 5HT used in these experiments already causes a >10-fold increase in egg laying (see Section 3.2), additional stimulation by LEV may have had little or no effect.

Why did only LEV-treated worms tend to slip through worm traps? We speculate that alterations in the shape and/or mechanical properties of worms’ bodies during spastic paralysis in LEV facilitate passage through the worm trap, whereas the flaccid paralysis caused by IVM or PIP does not do so. It would be informative to video-record worms during drug treatments to monitor body shape and movement within the worm trap, but this was beyond the scope of the present study. Redesigning chips with a smaller-diameter worm trap is possible, but hydrodynamic resistance to perfusate flow must be carefully balanced between the worm trap and side channels (Lockery et al., 2012).

To be included in EPG analysis (pump frequency, IPI, etc.), a worm's signal had to be recorded for 60 min post-switch; any worms expelled during this period were excluded from analysis. If paralyzed worms were the strongest responders to LEV, the concentration-response analysis of pump frequency and other parameters (Fig. 5, Fig. 7) may have underestimated drug potency.

### 3.7 Piperazine (PPZ) effects on susceptible and resistant *C. elegans*

The final anthelmintic drug tested was PPZ, a weak agonist of γ-aminobutyric acid receptors (GABA-Rs). GABAergic motoneurons inhibit body wall and head muscles, and excite enteric muscles, but there is no GABAergic innervation or GABA-Rs in the pharynx (Jorgensen, 2005). As an anthelmintic treatment, PPZ kills worms by inducing flaccid paralysis (Martin, 1985). The inhibitory GABA-R in *C. elegans* derives from *unc-49,* which is alternatively spliced into multiple subunits to form a heteromeric receptor (Bamber et al., 1999). We compared the responses of N2 worms with *unc-49* worms, as another opportunity to test the ability of EPG recordings to distinguish susceptible and resistant strains, and detect anthelmintic activity in a drug acting outside the pharynx.

Fig. 9Ai,ii show representative EPG recordings of N2 and *unc-49* worms before and after a switch from M9-5HT to M9-5HT (control experiments), which had no apparent effect on EPG activity. Fig. 9Aiii,iv compare N2 and *unc-49* worms after switching to 100 mM PPZ; pump frequency in the N2 worm decreased, in part from more frequent gaps, whereas pumping continued steadily in the *unc-49* worm, consistent with a PPZ-resistant phenotype. Before proceeding further, it is necessary to discuss the use of unusually high concentrations of PPZ to obtain responses within the 60-min post-switch period. In addition to potential contributions of cuticle impermeability, PDMS drug absorption and strong pumping stimulation by 10 mM 5HT, other studies show that PPZ has intrinsically low anthelmintic activity against *C. elegans*. Miltsch et al. (2013) reported that concentrations up to 100 mM PPZ failed to completely paralyze N2 worms and Racz et al. (2017) reported that a 72 h exposure to 10 mM PPZ failed to cause 100% lethality. In a 7-day treatment protocol, nearly 100% of worms were killed by 100 μg/ml (490 μM) LEV whereas only 15.5% were killed by 100 μg/ml (1.16 mM) PPZ (Gnoula et al., 2007). UNC-49 (GABA) channels from the parasitic nematode *Haemonchus contortus*, expressed in frog oocytes, require PPZ concentrations up to 100 mM for full activation, consistent with weak agonist activity even in the absence of a cuticular barrier (Brown et al., 2012). Thus, it is unsurprising that millimolar concentrations of PPZ were required for our experiments. We maintained physiological pH in PPZ working solutions with HEPES buffer, and used the same HEPES concentration in control perfusate (see Section 2.3).

Fig. 9B and C displays raw pump frequency data from N2 and *unc-49* worms, respectively, before and after switching to different concentrations of PPZ. Baseline pump frequency differed significantly between the two strains (N2, 3.98 ± 0.08 Hz, *n* = 97 worms; *unc-49*, 4.32 ± 0.08 Hz, *n* = 84 worms; *P* < 0.001). Normalized data in Fig. 9D show the reduced response to PPZ in *unc-49* worms, notably in the 40 and 100 mM PPZ groups. This effect was confirmed in CF plots (Fig. 9E); *CF*_50_ values in N2 and *unc-49* worms did not differ significantly in the control (*P* = 0.60) or 10 mM PPZ (*P* = 0.51) groups, but did so in the 40 mM (*P* < 0.002) and 100 mM (*P* < 0.01) groups. These data demonstrate a PPZ-resistant phenotype of *unc-49*, using microfluidic EPG recordings. Much less is known about anthelmintic resistance in *unc-49* mutants compared to mutations in GluCl or L-AChR genes. In a motility assay, Miltsch et al. (2013) reported that N2 and *unc-49* worms were equally affected by PPZ.

Fig. 5Aiii shows IPI data for N2s treated with PPZ. The mode of the IPI distribution was similar in all groups (range, 195–225 ms; 5.1 to 4.4 Hz) with no consistent relationship between mode and concentration. The right shoulder of the distribution increased with increasing PPZ concentration; the 0 and 10 mM histograms were similar, while 40 and 100 mM PPZ showed progressively greater shifts toward higher probability of longer IPIs. The IPI histogram for PPZ much more closely resembled that of IVM (Fig. 5Ai) than LEV (Fig. 5Aii). Fig. 5B shows that, for an intermediate PPZ (40 mM) in N2 worms, pump duration was relatively stable with perhaps some increase toward the end.

In summary, high concentrations of PPZ (40 and 100 mM) inhibited pumping in a concentration-dependent manner, with *unc-49* showing decreased sensitivity to the drug. PPZ, like LEV, targets receptors located outside of the pharynx, respectively activating inhibitory GABA-Rs and excitatory L-AChRs on body wall muscles. Similarly to IVM, which also causes flaccid paralysis, PPZ did not cause a significant increase in the percentage of worms expelled through the worm trap (see Section 3.6). Our data confirm the ability of EPG recordings to detect extra-pharyngeal anthelmintic actions in two classes of anthelmintic drugs with different targets.

## 4 Conclusions

Many anthelmintic drugs act on ICs/NTRs, which control the electrical activity of neurons and muscles, and these proteins remain valuable targets for anthelmintic drug development. To facilitate the use of electrophysiological recordings in anthelmintic research, we developed a microfluidic EPG platform that collects continuous EPG recordings from 8 worms simultaneously while perfusing test solutions (Lockery et al., 2012). In the present study, we improved the chip design and developed improved algorithms for spike recognition and data analysis. We had two main experimental objectives. The first was to optimize methods for stimulating robust, sustained pharyngeal pumping in *C. elegans* in microfluidic EPG chips. Sections 3.2, 3.3 describe the concentration dependence and temporal characteristics of pumping induced by the neuromodulator 5HT and a more natural stimulus, *E. coli* bacteria. We chose to use 10 mM 5HT as the pumping stimulus against which to test inhibitory effects of anthelmintic drugs (Sections 3.4 to 3.7) but the features of OP50-stimulated pumping make it a suitable option as well. The lower pump frequencies achieved in OP50 should not interfere with anthelmintic detection (Weeks et al., 2016b).

The second objective was to further validate the use of EPG recordings and automated data analysis by characterizing the effects on pharyngeal pumping of three representative anthelmintic drugs that act on ICs/NTRs: specifically, we examined the effects of IVM on GluCl, LEV on L-AChRs and PPZ on GABA-Rs (Sections 3.4 to 3.7). The latter two drugs act outside the pharynx but inhibited pumping, validating EPG recordings as a readout for drugs acting within or outside the pharynx. Similarly, pharyngeal pumping is used as a proxy for the general health status of *C. elegans* in studies of aging, toxicology and other stressors (e.g., Yu and Driscoll, 2011; Russell et al., 2017; Xu et al., 2017). For all three anthelmintic drugs tested, EPG recordings successfully distinguished susceptible from resistant strains of *C. elegans*.

Continuous EPG recordings taken before and during drug treatment provide exceptional statistical power. A typical control experiment in 10 mM 5HT, with a worm pumping at ∼4.5 Hz for 90 min, yields data on > 24,000 individual pumps; in the 8-channel EPG platform, this represents almost 200,000 pumps acquired over 90 min. A typical sample size of 20 worms in an experimental group yields almost 4 million pumps for automated analysis. Furthermore, because EPGs are recorded both before and after drug exposure in each worm, within-subjects statistical tests can be employed. The parameters analyzed in this study—pump frequency, pump duration and IPI—are only a subset of potentially informative features that can be mined from EPG recordings. The phenotypes produced by anthelmintic drugs are not especially subtle, but the ease of rapidly collecting large EPG data sets for statistical analysis facilitates identification of subtle pumping phenotypes that would be missed by traditional methods such as visually counting pumps. The throughput of the current 8-channel EPG platform could be increased by adding more recording modules per chip or running multiple chips in parallel. Importantly, the 8-channel EPG platform is compatible with a diverse range of nematode species: *C. elegans* (Lockery et al., 2012; Weeks et al., 2018), other free-living nematodes (Weeks, 2016) and host-stage hookworms and ascarids (Weeks et al., 2016b).

The microfluidic device used here is one of many that have recently become available for studies of anthelmintics and drug resistance. The devices fall into two main classes. The first class comprises devices for characterizing locomotion, including both crawling and swimming. This is done in straight channels or arena-shaped chambers in response to anthelmintics such as LEV (Carr et al., 2011; Chen et al., 2011; Liu et al., 2012, 2013; Lycke et al., 2013; Ding et al., 2017). In an exceptional case, worms were enclosed in aqueous droplets (Aubry and Lu, 2017). Locomotion parameters, including speed, undulation frequency, and body shape, are extracted from video recordings or, in one instance, from resistance measurements across an electrode array (Liu et al., 2013). The second class of microfluidic devices, such as the one used here, make electrical recordings of muscle and neuron activity, including pharyngeal contractions (Lockery et al., 2012; Hu et al., 2013; Weeks et al., 2016b) and movements of the stylus in the case of plant nematodes (Hu et al., 2014). A microfluidic device that records electrical potentials from *C. elegans* body wall muscles (Gonzales et al., 2017) has not, to our knowledge, been applied to anthelmintic research.

There are advantages and disadvantages to each microfluidic platform. Locomotion, like electrical recordings, provides a readout of neuromuscular activity and worm health. Locomotion can generally be measured at higher throughput than for existing electrical recording devices, and a large literature on anthelmintic effects on worm movement in non-microfluidic conditions is available for comparison. A disadvantage of locomotion assays are that they are agnostic in suggesting specific targets or mechanisms of action of candidate drugs, although mutant analysis in *C. elegans* can help with this. The higher throughput and non-specificity of locomotion assays can be advantageous for primary screens, whereas lower-throughput secondary screening using electrical recordings can suggest specific ICs or NTRs that may underlie a drug effect. For example, in a screen of the NIH Clinical Collection library for anthelmintic candidates, primary screening was accomplished using development, lethality and locomotion assays, followed by EPG analysis for more detailed characterization (Weeks et al., 2018). Ultimately, drugs acting on ICs/NTRs can be investigated in precise detail by electrophysiological methods such as voltage clamp recordings in situ or in heterologous expression systems (e.g., Vinogradova et al., 2006; Robertson and Martin, 2007; Abongwa et al., 2016).

For microfluidic EPG recordings, a microfluidic chip closely related to ours, called the NeuroChip, was introduced in 2013 (Hu et al., 2013). Both systems enable drug perfusion during EPG recordings and offer automated event-recognition software (Dillon et al., 2009) but have unique and complementary strengths. In the NeuroChip, the shape and materials of the worm trap are optimized to detect synaptic events in EPG waveforms and the chip incorporates a sorting system for selecting individual worms based on their electrical signatures. It is possible to recover worms from the 8-channel chip after EPG recordings, but this is inconvenient (see Section 3.4). Strengths of the 8-channel platform include the increased throughput afforded by multiple simultaneous recordings and an enhanced spike detection algorithm; AutoEPG software detects R spikes using a fixed threshold and E spikes are detected as the highest voltage within a fixed time window prior to the R spike (Dillon et al., 2009). This approach is problematic for long recordings during which EPG waveform shapes and amplitudes change over time, e.g., in response to anthelmintic drugs. To surmount this difficulty, our software employs automatic gain control and optimizes six parameters that gave the most reliable pump detection, as evidenced by maximizing a fitness function (see Section 2.6). Finally, the NeuroChip and 8-channel platform offer different advantages depending on the duration of EPG recordings desired. Once loaded with worms, the 8-channel chip provides stable EPG recordings for >90 min (this study) and potentially for 6–8 h (Lockery et al., 2012), which is useful for determining the time course of slowly-acting drugs. In contrast, the NeuroChip uses a valve system to position worms for recording, one at a time, in succession. A single-channel system developed from the original 8-channel platform, called the ScreenChip, is commercially available and likewise provides rapid sequential EPG recordings (e.g., Russell et al., 2017), but does not offer perfusion or worm sorting. New and improved microfluidic devices should continue to become available from academic and commercial laboratories.

In summary, the results obtained in the current study should provide a useful framework for investigators who would like to adopt electrophysiology into their research workflow for anthelmintic drug screening, drug resistance, basic research on nematode feeding behavior, and other aspects of nematode biology.

## Conflicts of interest

The authors declare the following potential competing financial interests: JCW, KJR, SRL and WMR own equity in NemaMetrix, Inc., which holds the sole commercial license for the patented microfluidic EPG device reported here.

## Author contributions

JCW and KJR designed the experiments and KJR performed them. WMR and JCW performed data and statistical analysis. SRL designed the microfluidic chip. JCW drafted the manuscript. All authors participated in editing the manuscript.
